# Supplementary material for: Comparative Studies of Fraxinus Species from Korea Using Microscopic Characterization, Phytochemical Analysis, and Anti-Lipase Enzyme Activity
Source: Plants (Basel). 2020 Apr 20;9(4):534. doi: 10.3390/plants9040534 (PMC7238101; doi:10.3390/plants9040534)
Supplement: Supplementary file 1 [file plants-09-00534-s001.pdf]

Supplementary Materials

Comparative Studies of *Fraxinus* Species from Korea Using Microscopic Characterization, Phytochemical Analysis, and Anti-lipase Enzyme Activity

Kazi-Marjahan Akter [1,†], Woo Sung Park [1,†], Hye-Jin Kim [1,†], Atif Ali Khan Khalil [1,2] and Mi-Jeong Ahn [1,*]

[1] College of Pharmacy and Research Institute of Pharmaceutical Sciences, Gyeongsang National University, Jinju 52828, Gyeongsangnam-do, Republic of Korea; marjahan7silva@gmail.com (K.-M.A.); pws8822@gmail.com (W.S.P.); black200203@gmail.com (H.-J.K.); atif.khalil7799@gmail.com (A.A.K.K.)
[2] Department of Biological Sciences, National University of Medical Sciences, 46000 Rawalpindi, Pakistan
* Correspondence: amj5812@gnu.ac.kr; Tel.: +82-55-772-2425
† These authors contributed equally to this work.

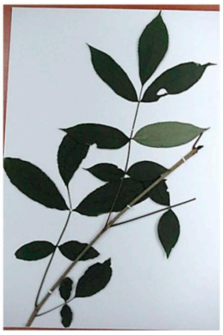

F. chiisanensis
PGSC-560

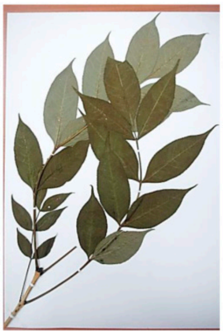

F. mandshurica
PGSC-561

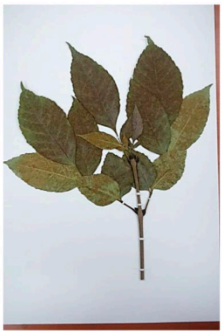

F. rhynchophylla
PGSC-562

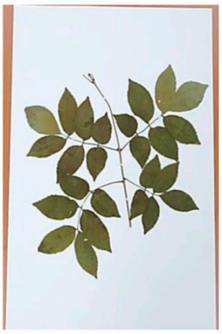

F. sieboldiana
PGSC-563

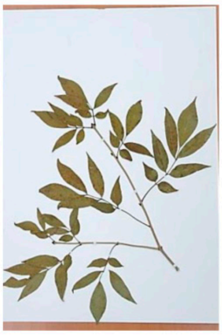

F. sieboldiana* var. *angustata
PGSC-564

Figure S1. Herbarium specimens of four *Fraxinus* spp. and one variety.

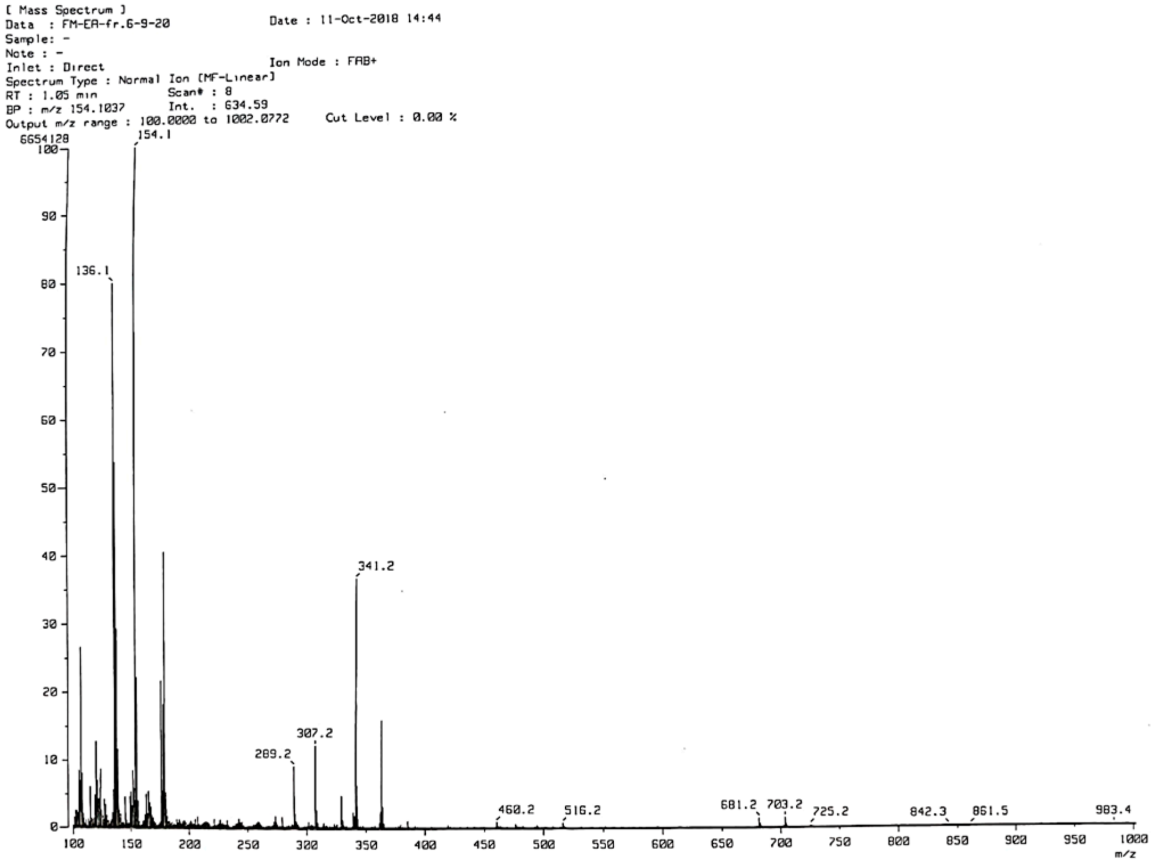

Figure S2. FAB-MS of compound **1** (*m*-NBA).

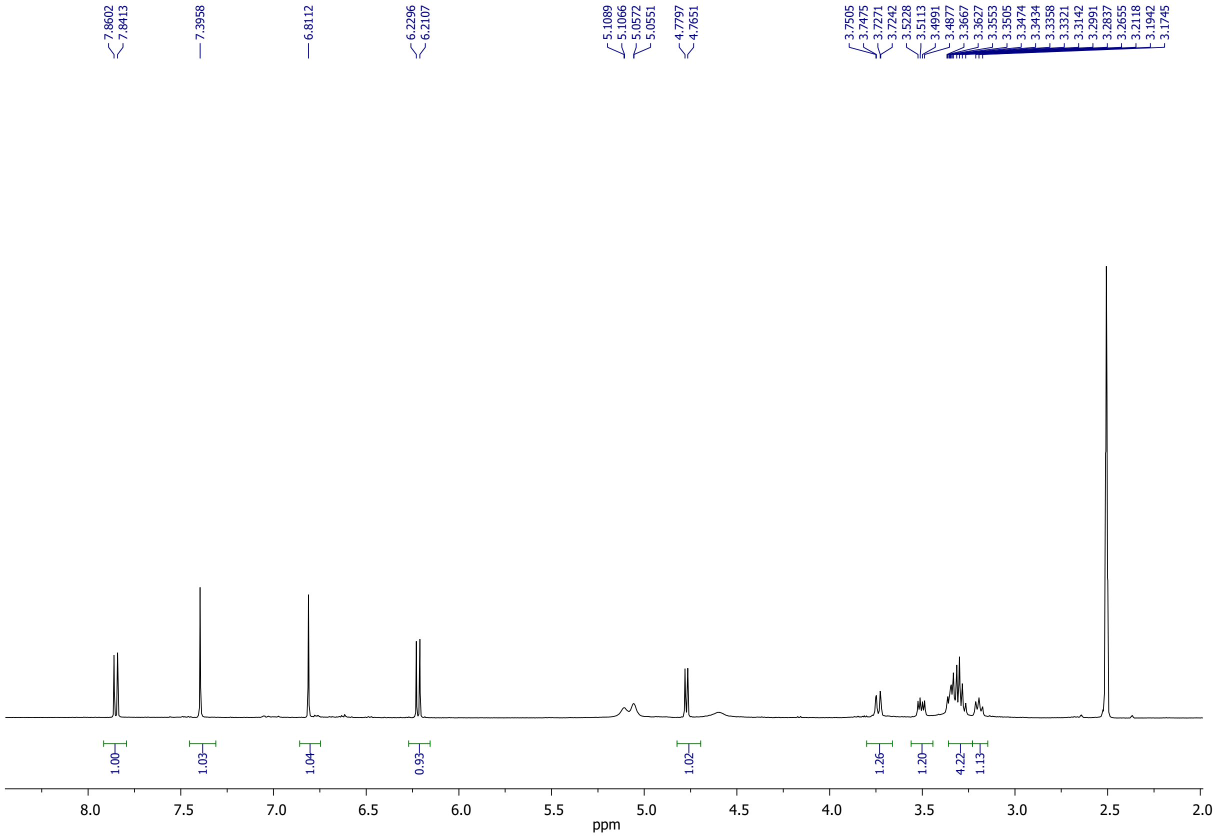

Figure S3. ^{1}H-NMR spectrum of compound **1** (500 MHz, DMSO-d_6).

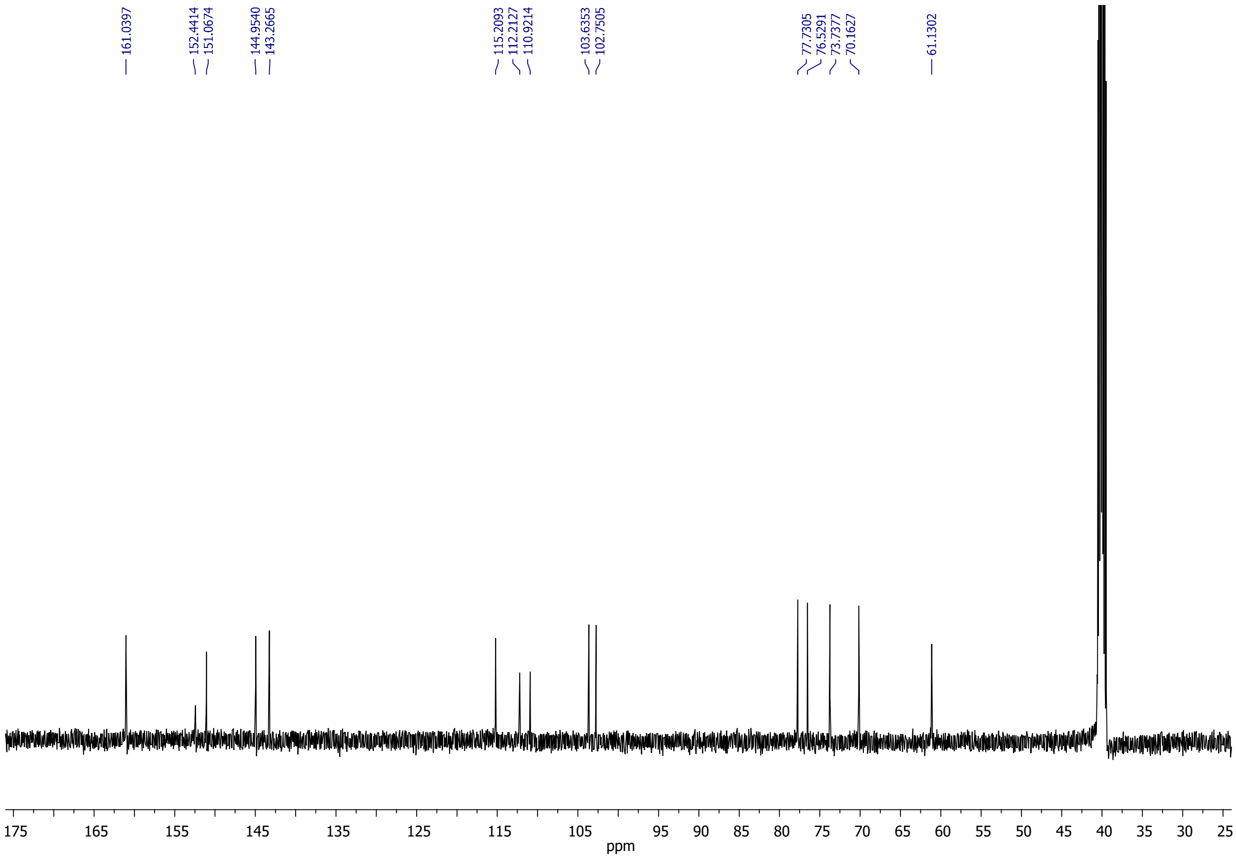

Figure S4. ^{13}C-NMR spectrum of compound **1** (125 MHz, DMSO-d_6).

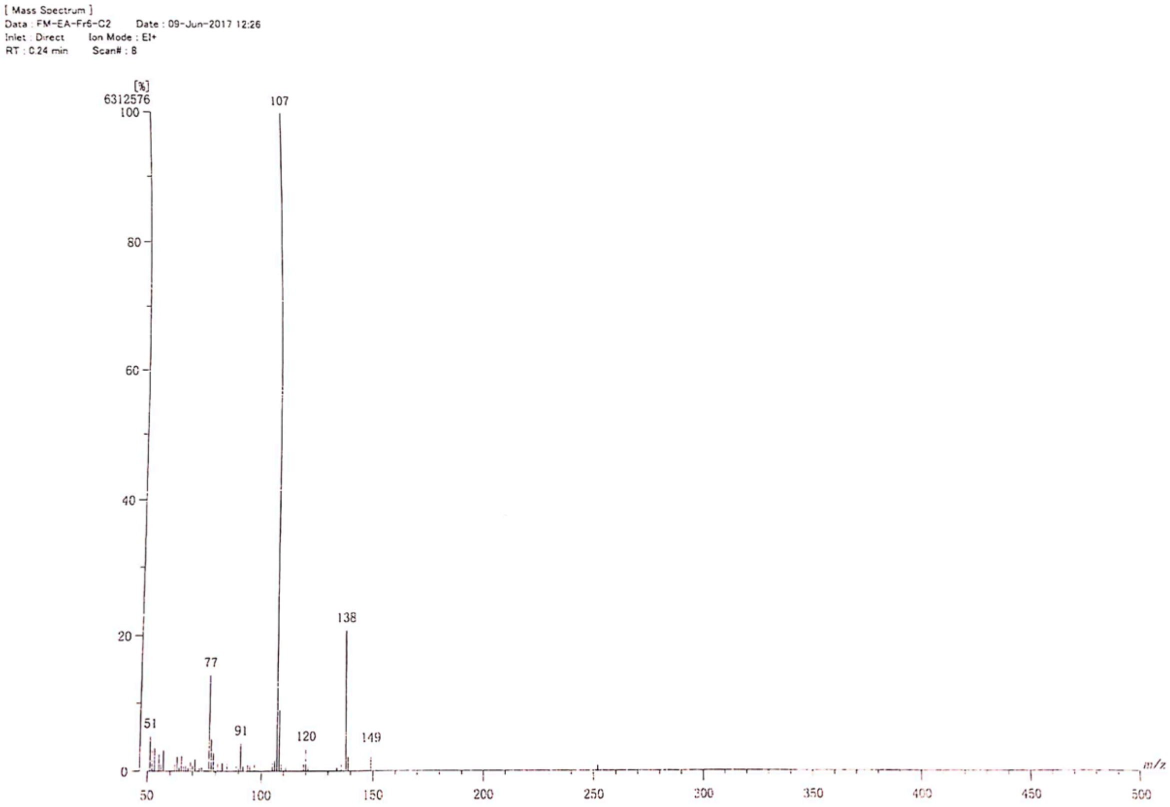

Figure S5. EI-MS of compound **2** (70 eV).

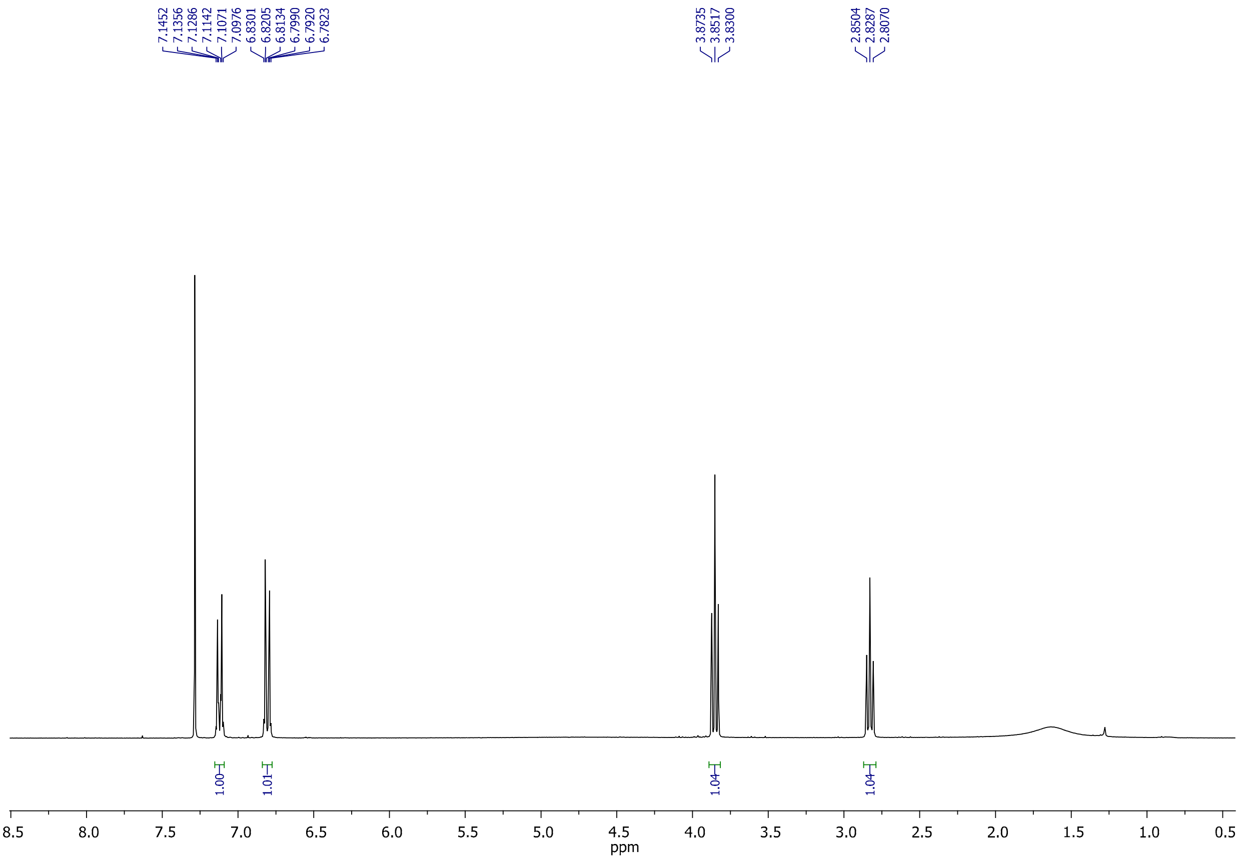

Figure S6. ^{1}H-NMR spectrum of compound **2** (300 MHz, $CDCl_3$).

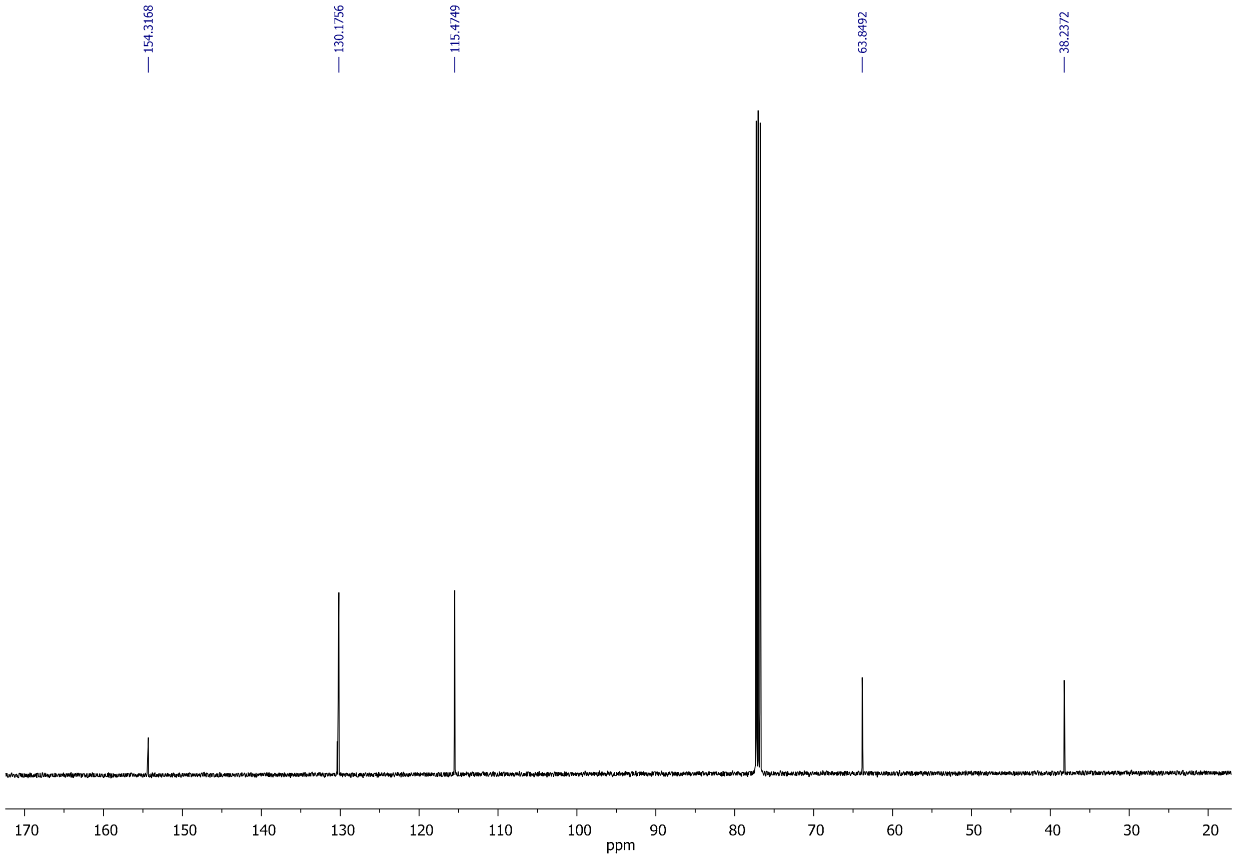

Figure S7. ^{13}C-NMR spectrum of compound **2** (125 MHz, $CDCl_3$).

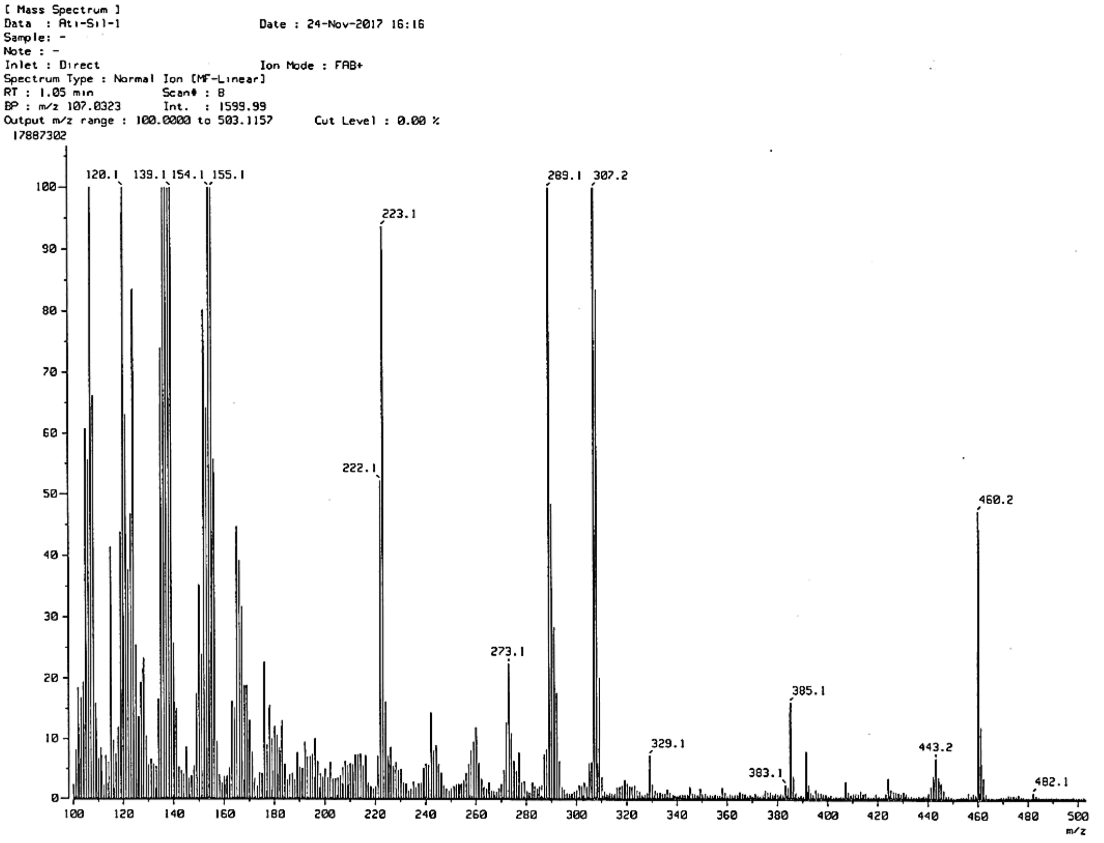

Figure S8. FAB-MS of compound **3** (*m*-NBA).

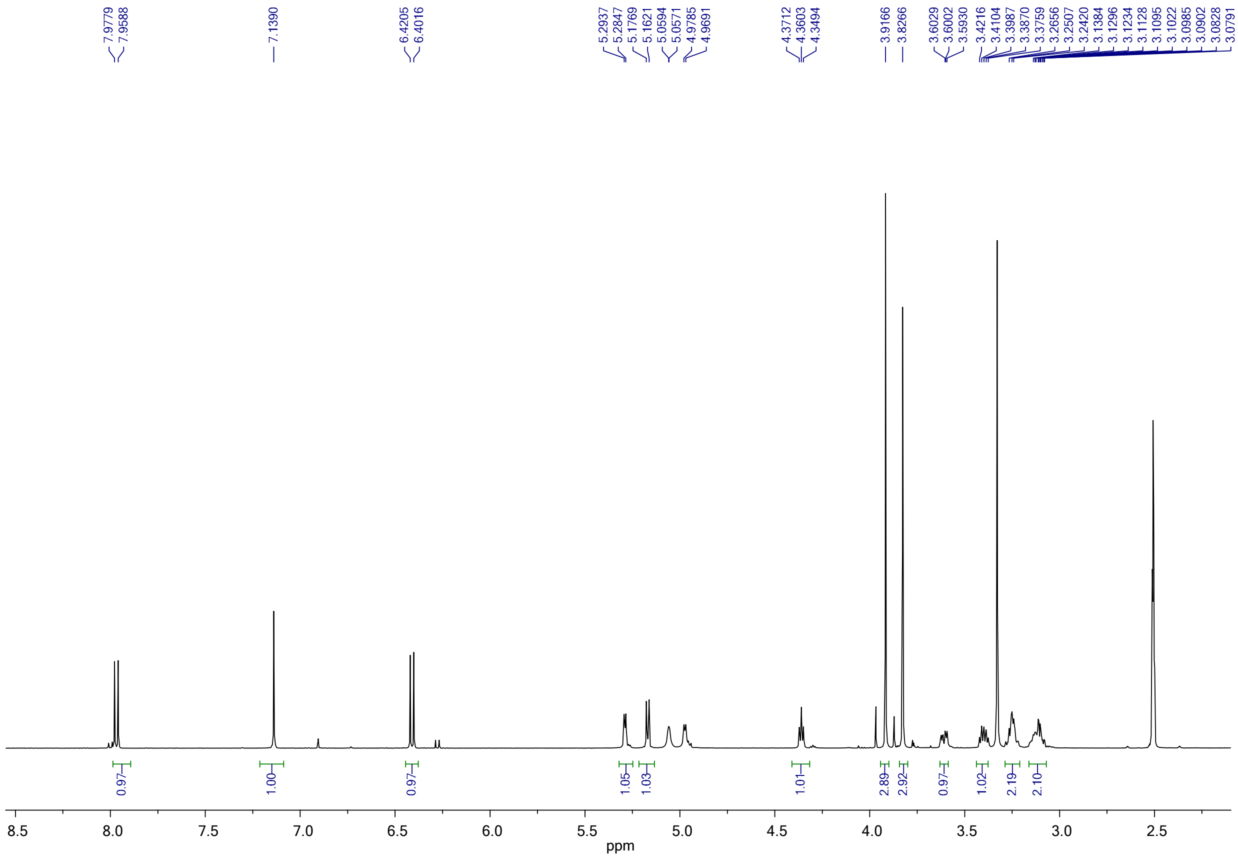

Figure S9. ^{1}H-NMR spectrum of compound **3** (500 MHz, DMSO-d_6).

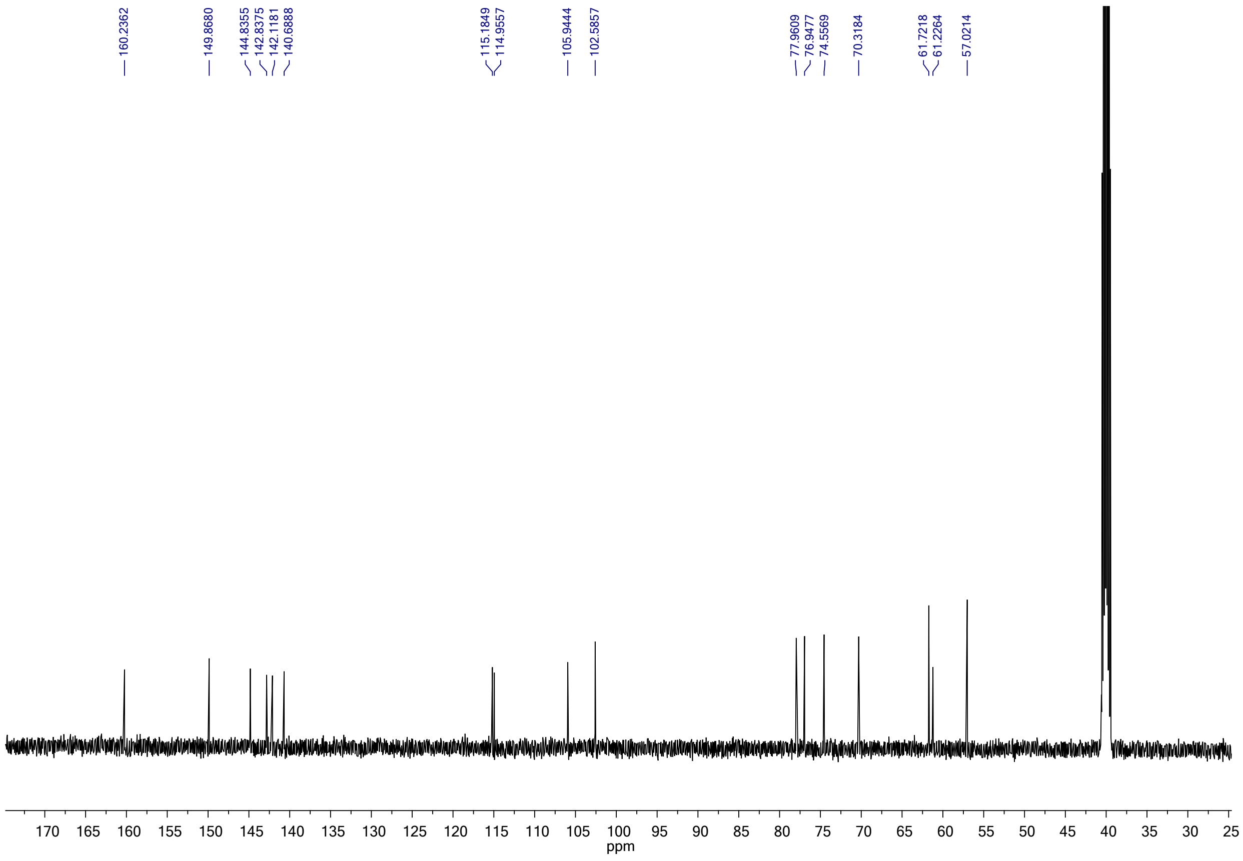

Figure S10. ^{13}C-spectrum of compound **3** (125 MHz, DMSO-d_6).

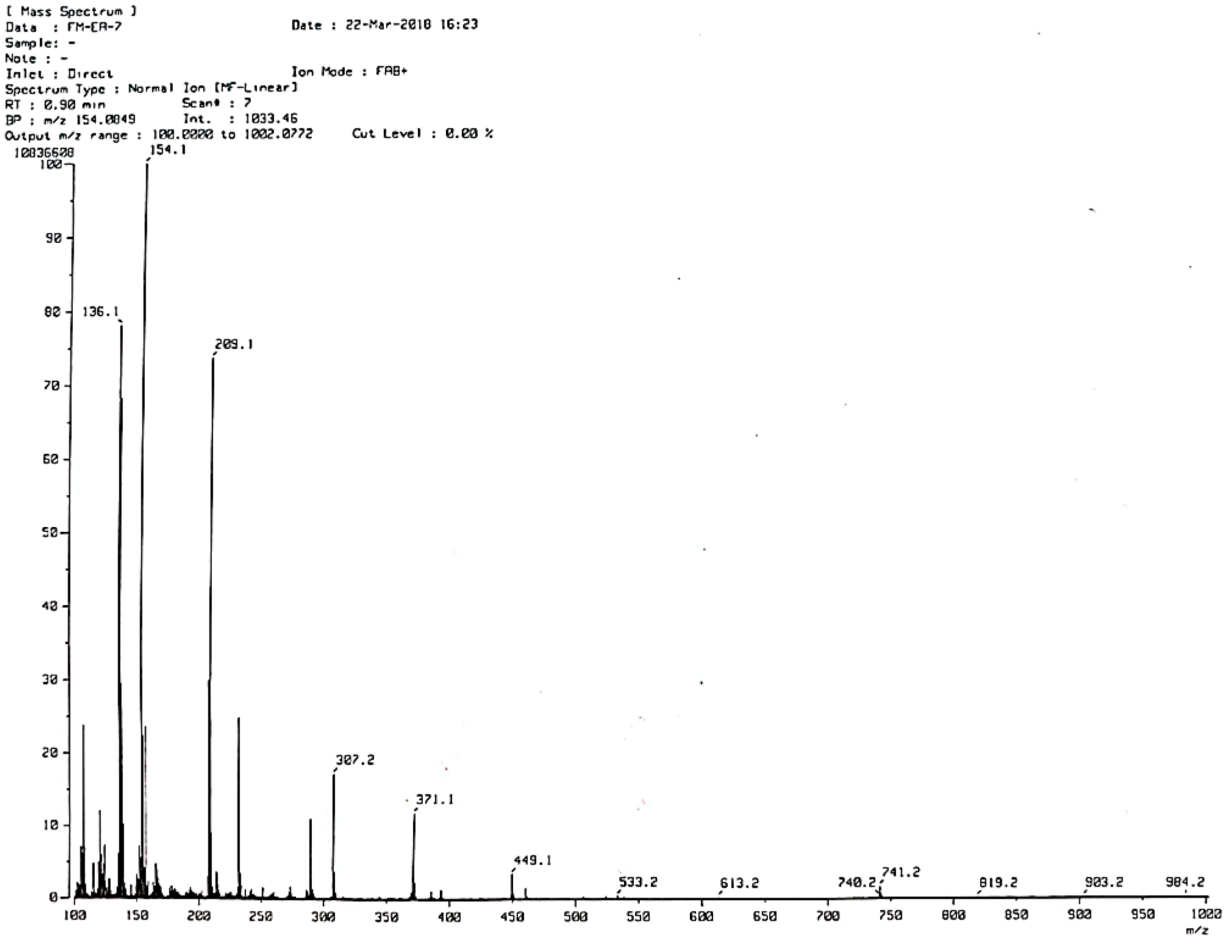

Figure S11. FAB-MS of compound **4** (*m*-NBA).

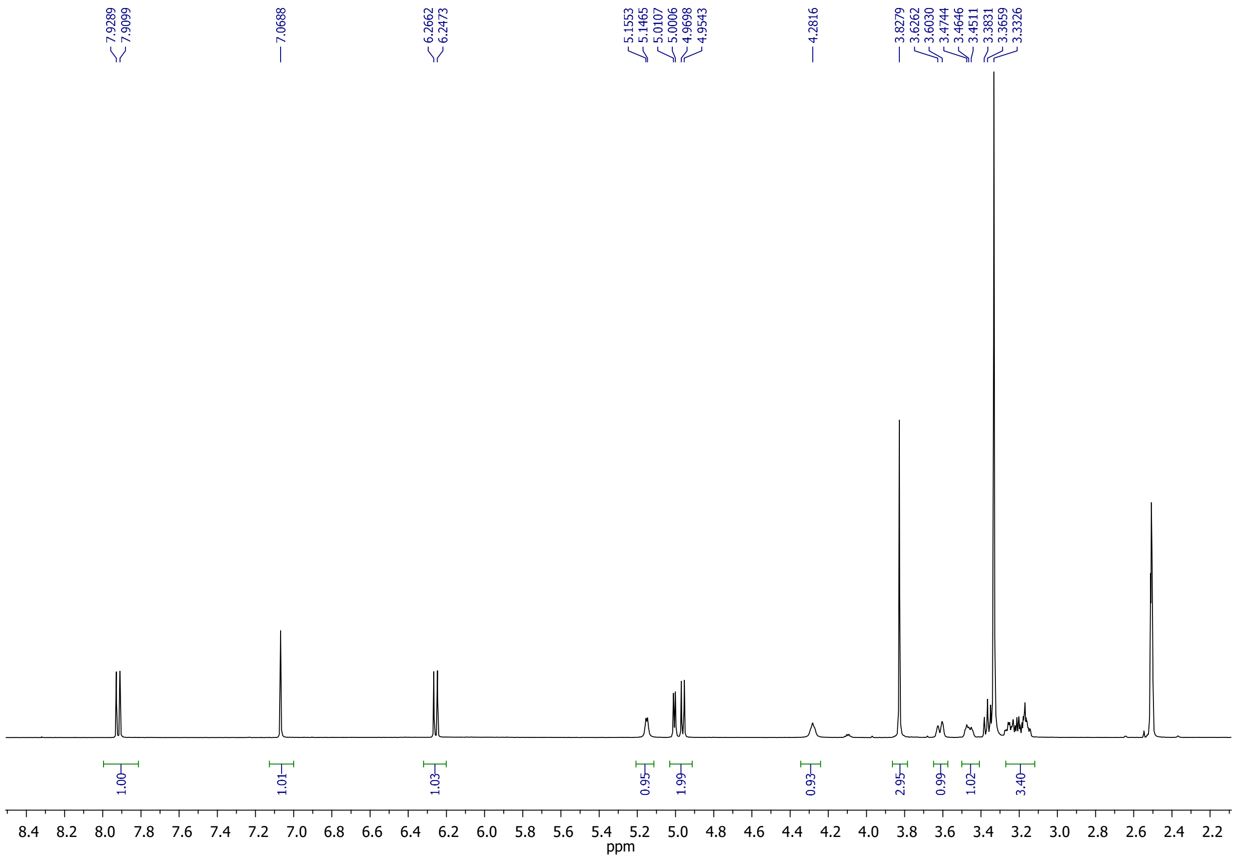

Figure S12. ^{1}H-NMR spectrum of compound **4** (500 MHz, DMSO-d_6).

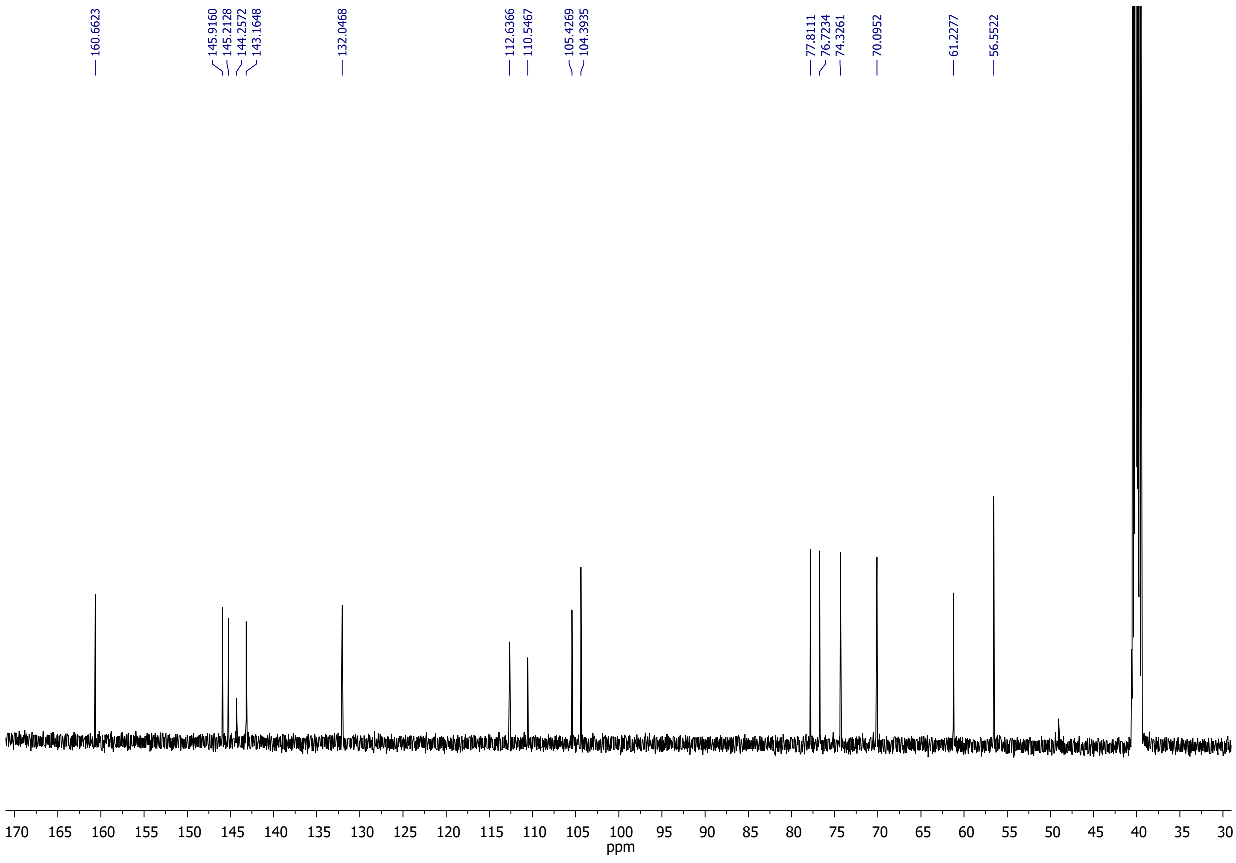

Figure S13. ^{13}C-NMR spectrum of compound **4** (125 MHz, DMSO-d_6).

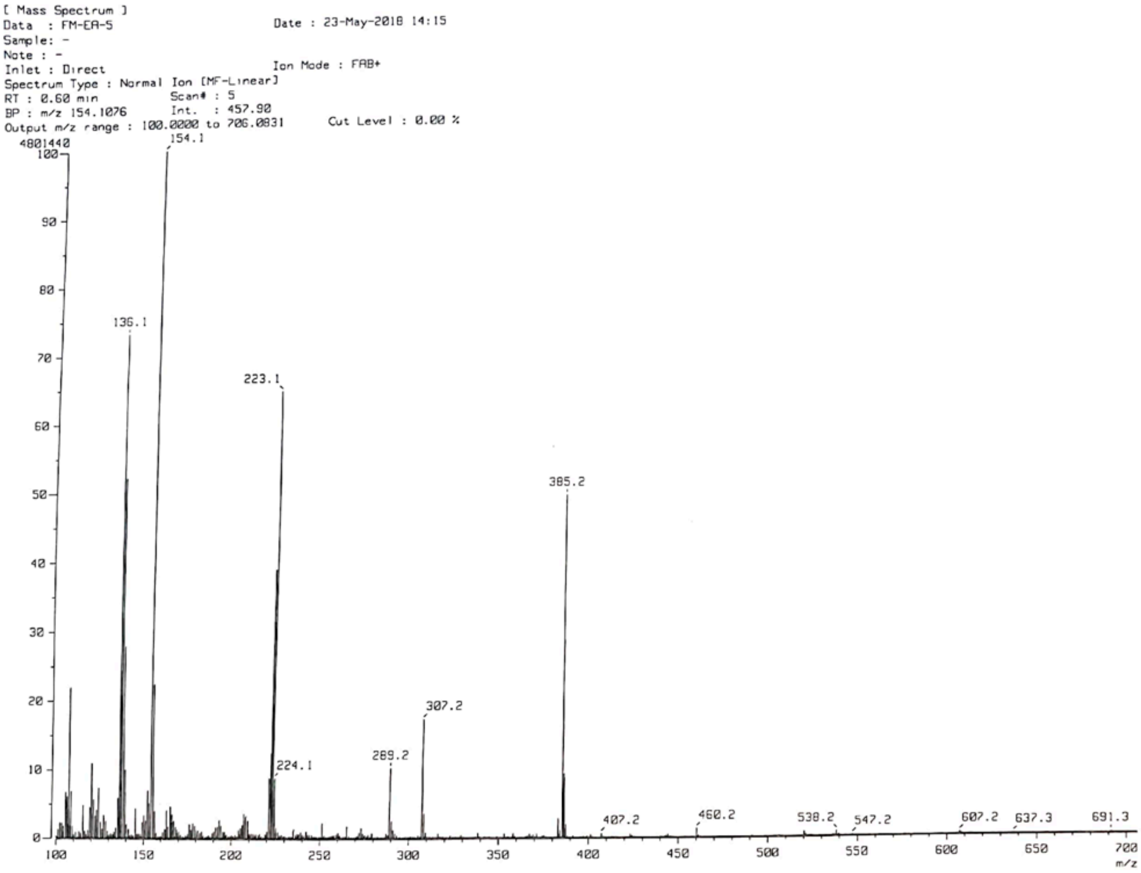

Figure S14. FAB-MS of compound **5** (*m*-NBA).

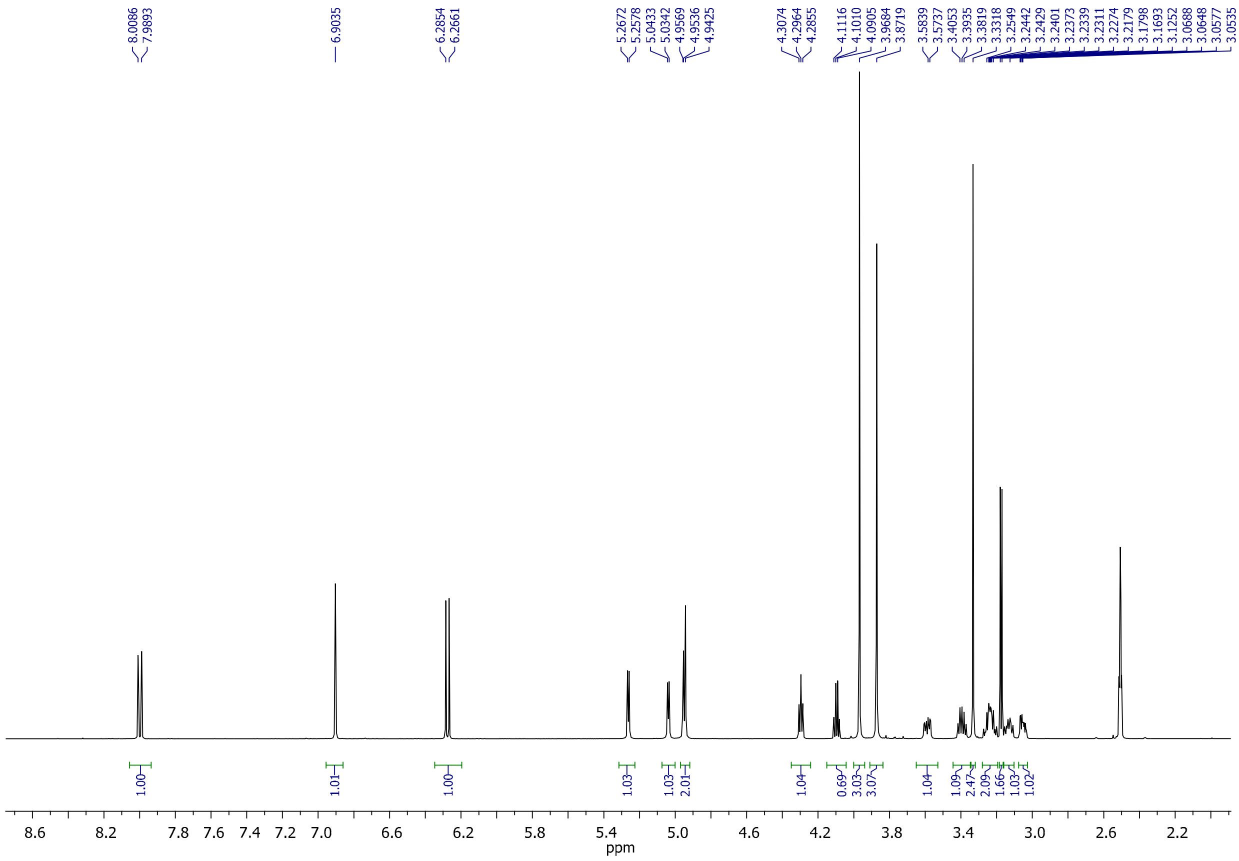

Figure S15. ^{1}H-NMR spectrum of compound **5** (500 MHz, DMSO-d_6).

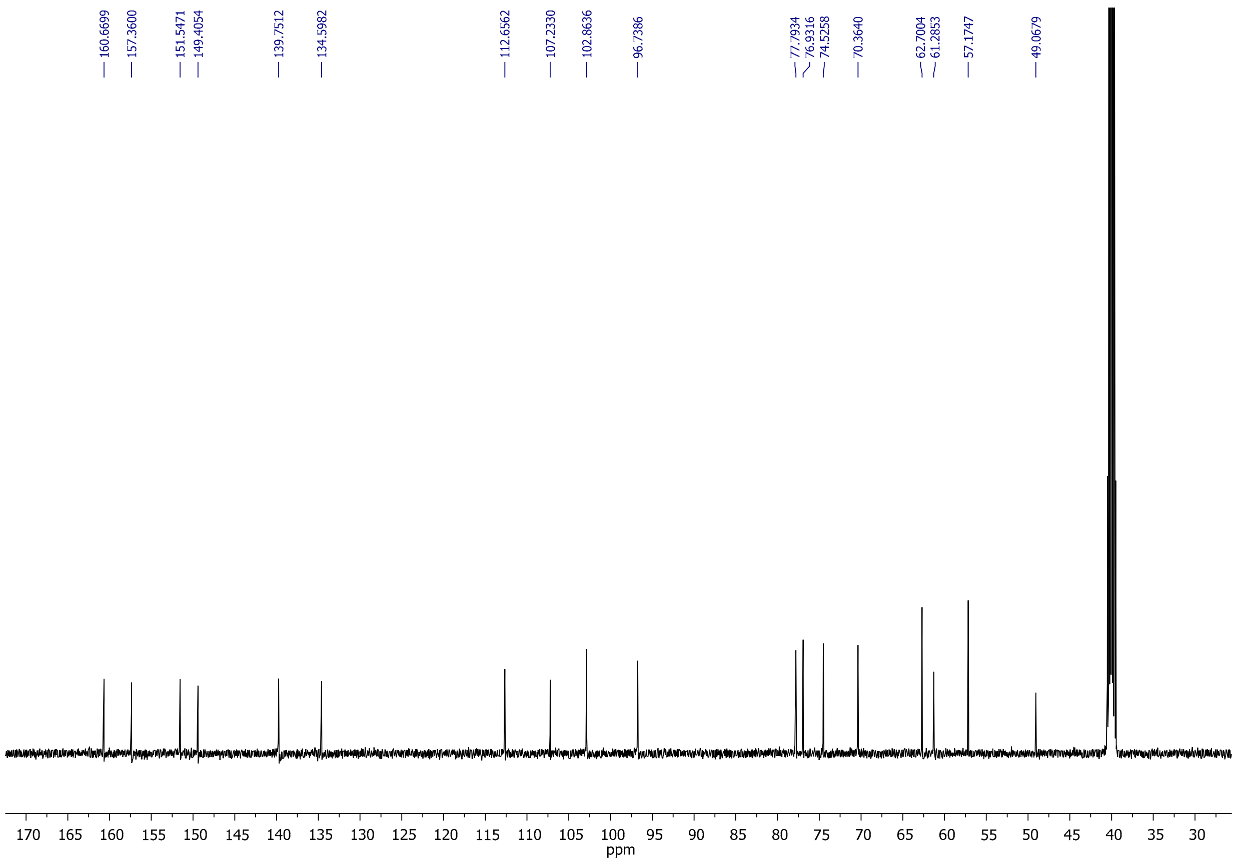

Figure S16. ^{13}C-spectrum of compound **5** (125 MHz, DMSO-d_6).

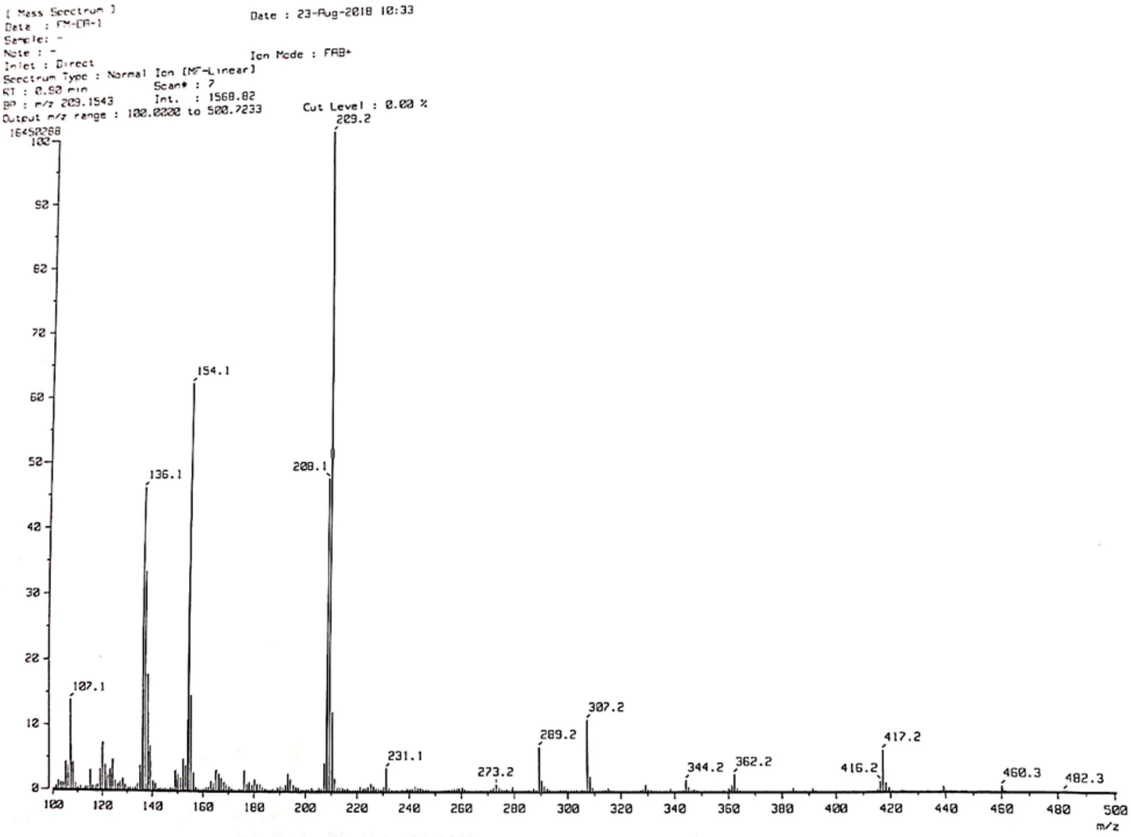

Figure S17. FAB-MS of compound **6** (*m*-NBA).

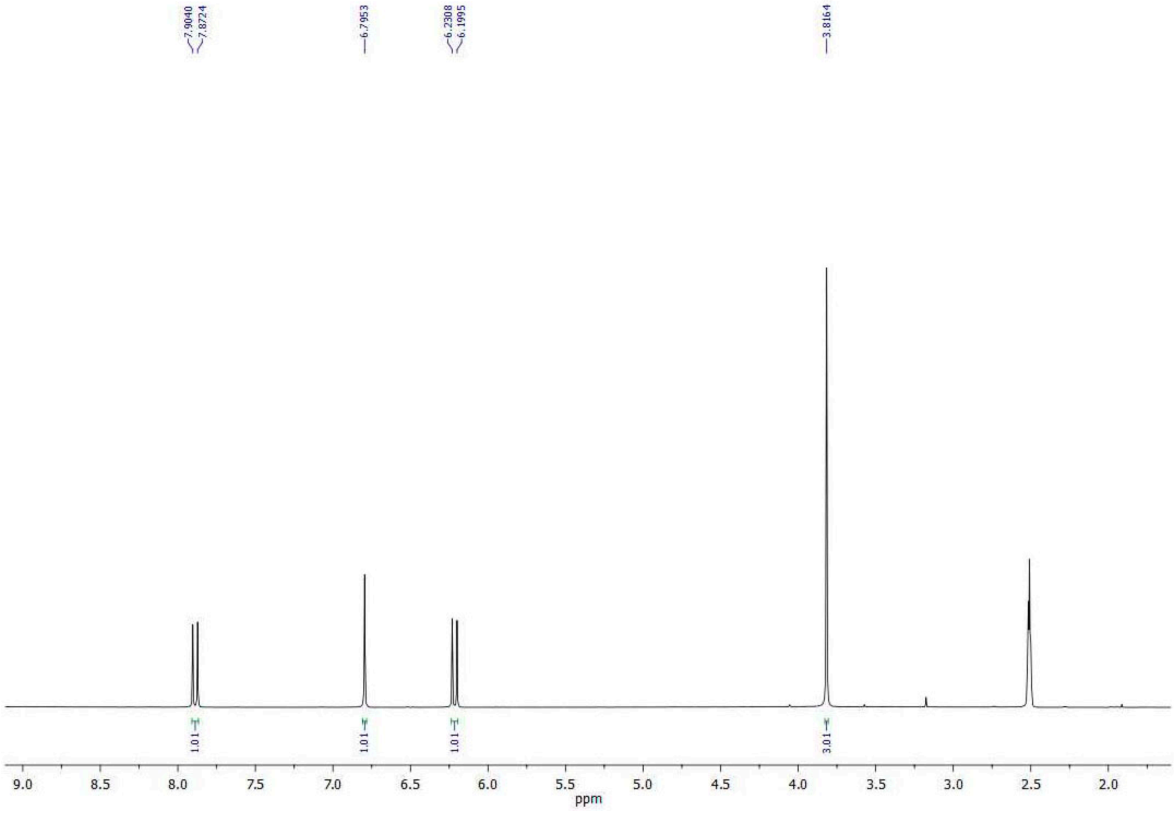

Figure S18. ^{1}H-NMR spectrum of compound **6** (300 MHz, DMSO-d_6).

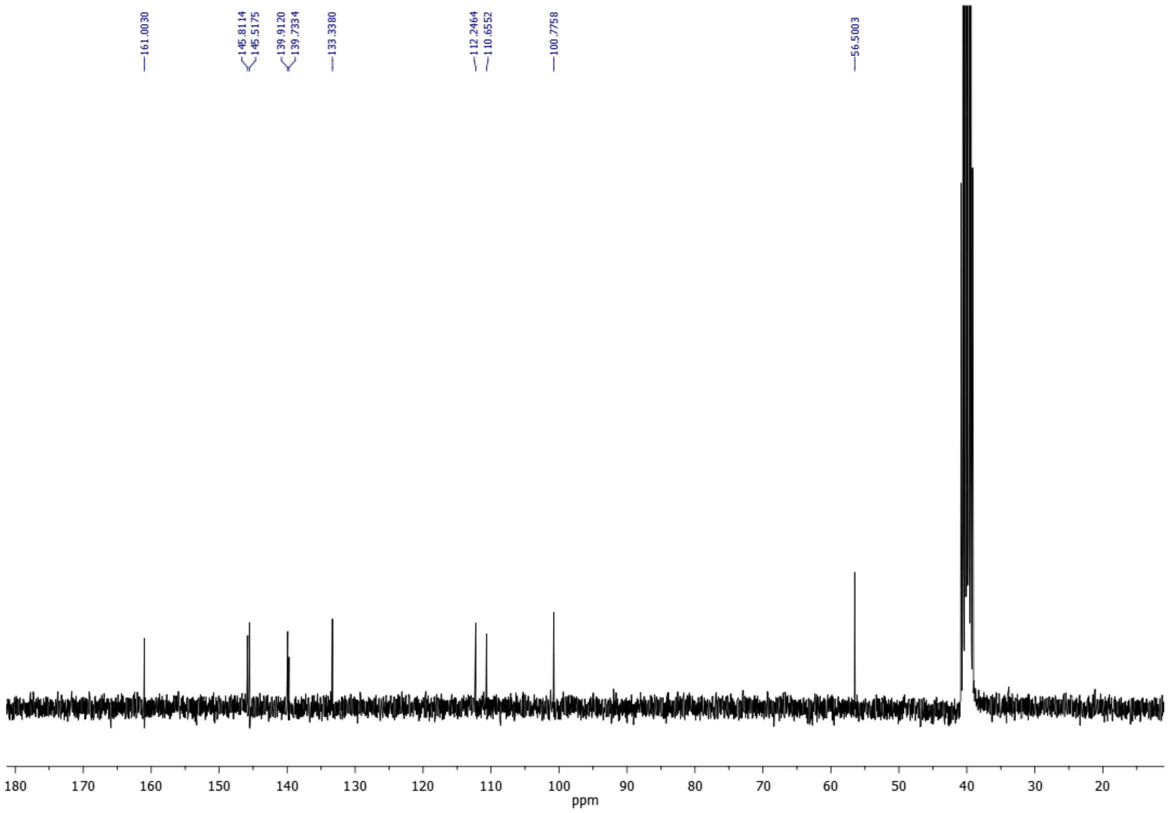

Figure S19. ^{13}C-NMR spectrum of compound **6** (75 MHz, DMSO-d_6).

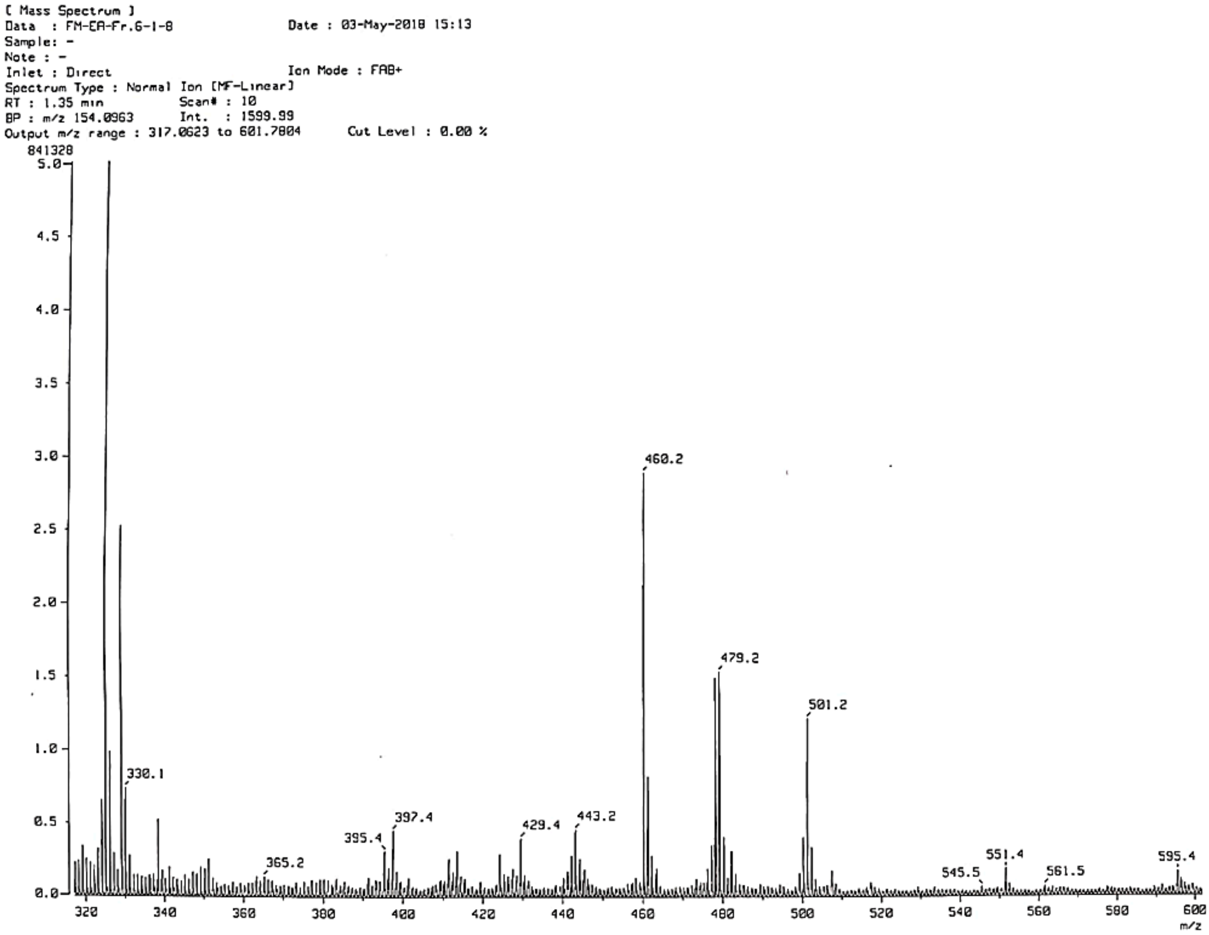

Figure S20. FAB-MS of compound **7** (*m*-NBA).

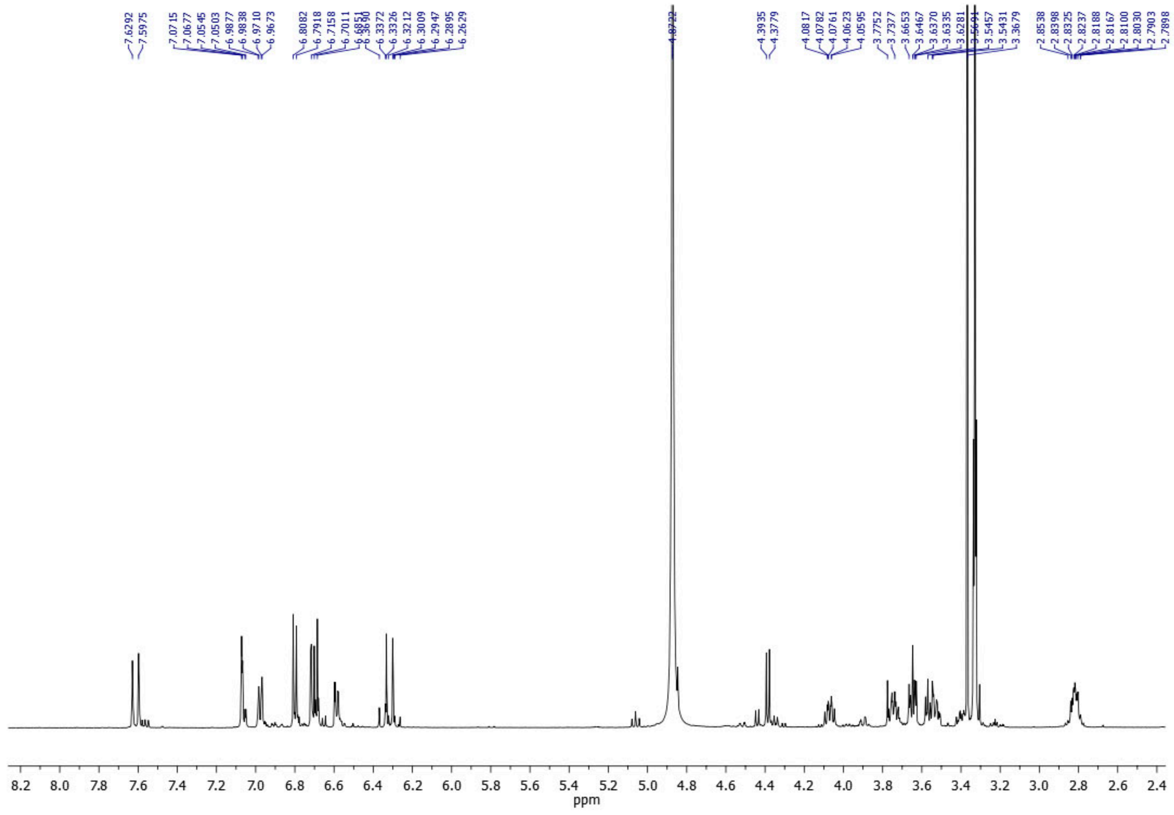

Figure S21. ^{1}H-NMR spectrum of compound **7** (500 MHz, CD_3OD).

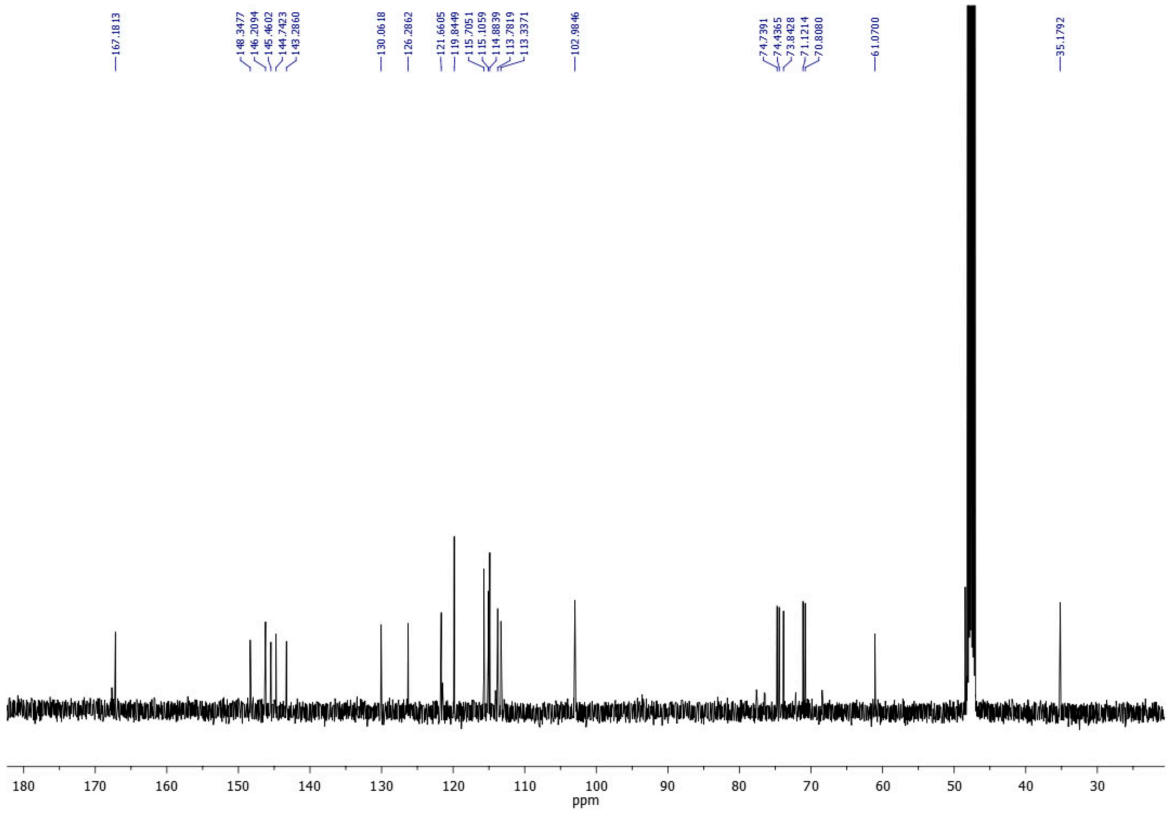

Figure S22. ^{13}C-NMR spectrum of compound **7** (125 MHz, CD_3OD).

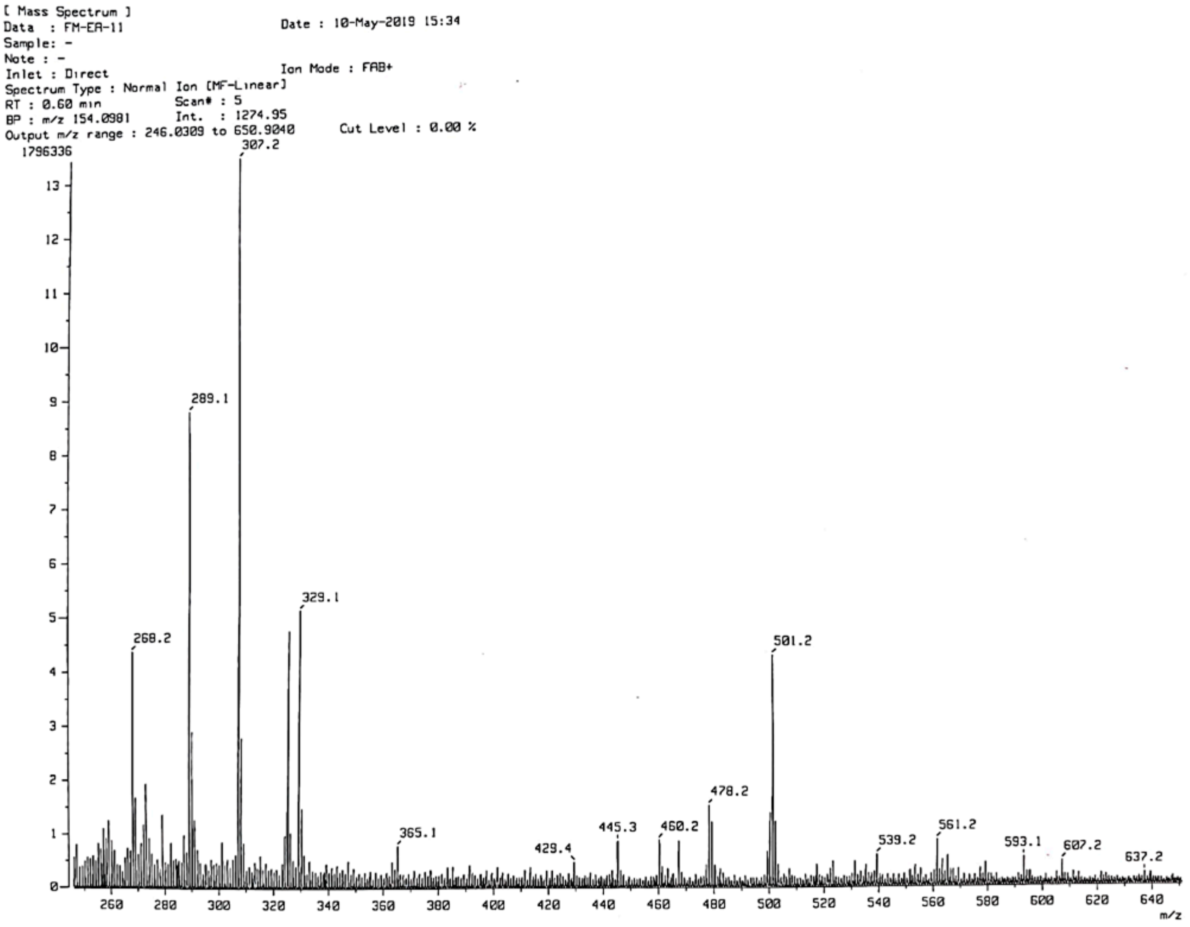

Figure S23. FAB-MS of compound **8** (*m*-NBA).

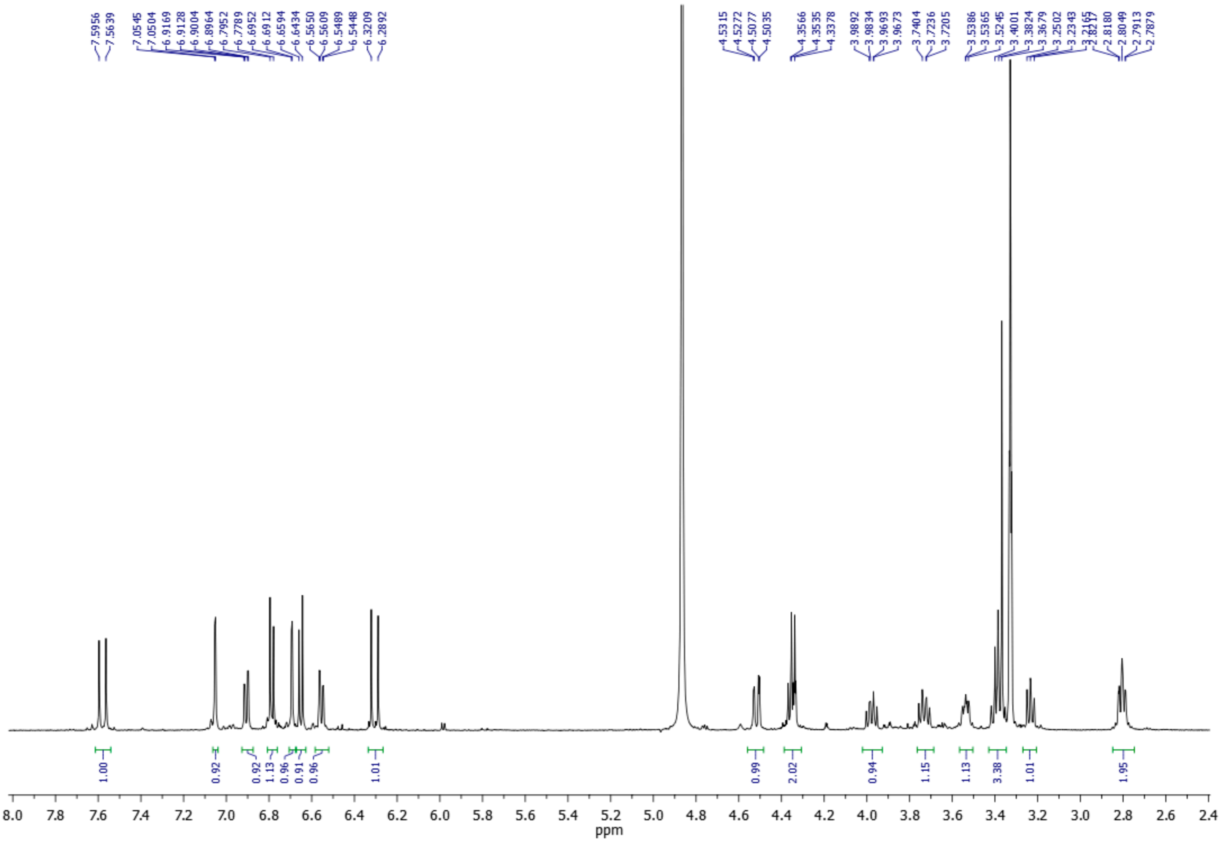

Figure S24. ^{1}H-NMR spectrum of compound **8** (500 MHz, CD_3OD).

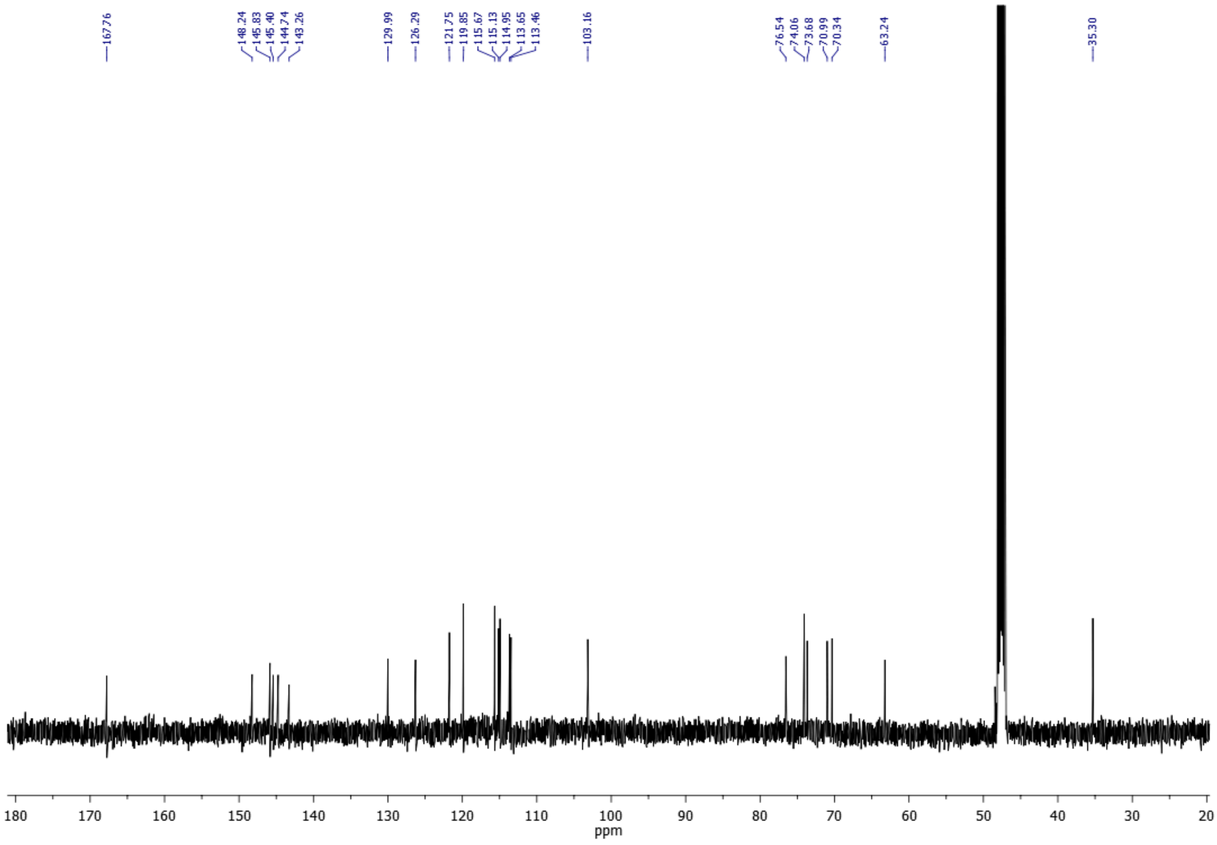

Figure S25. ^{13}C-NMR spectrum of compound **8** (125 MHz, CD_3OD).

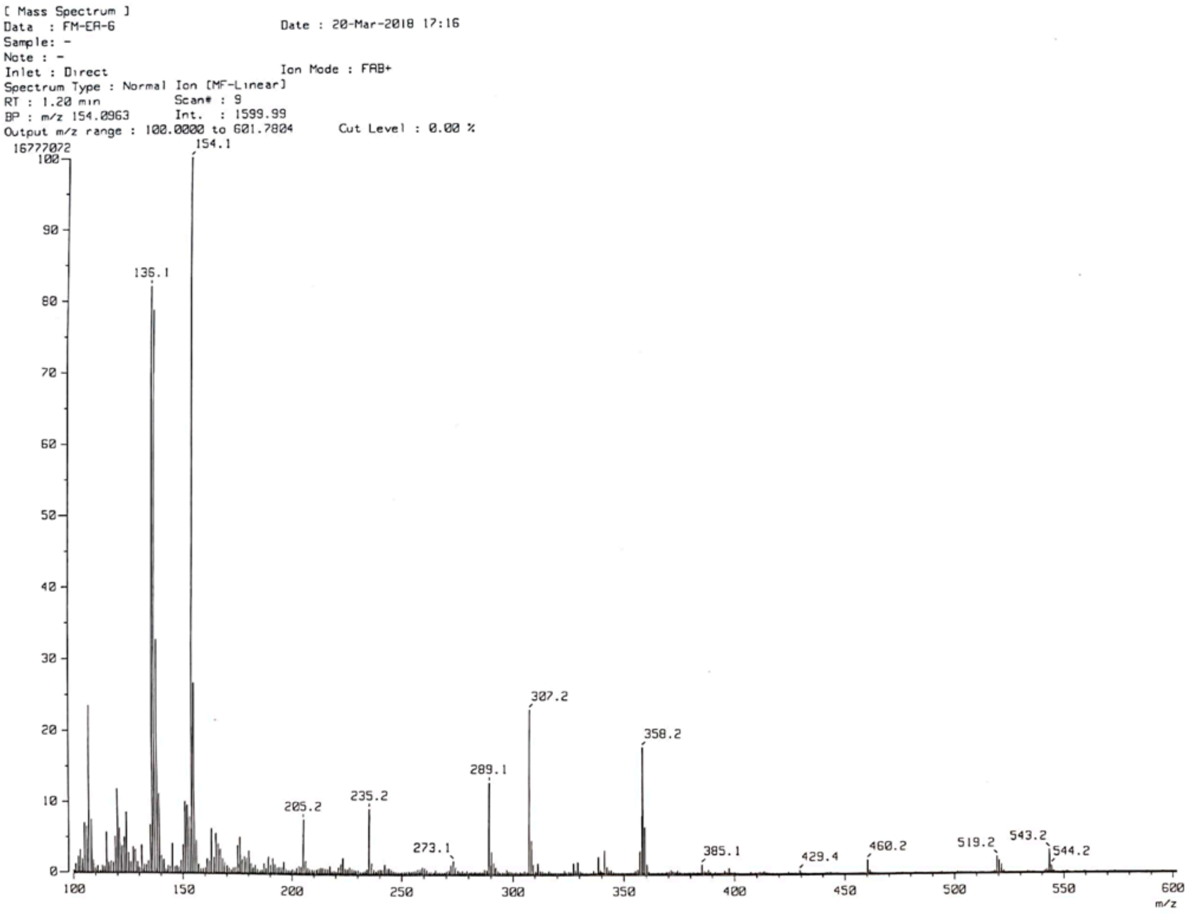

Figure S26. FAB-MS of compound **9** (*m*-NBA).

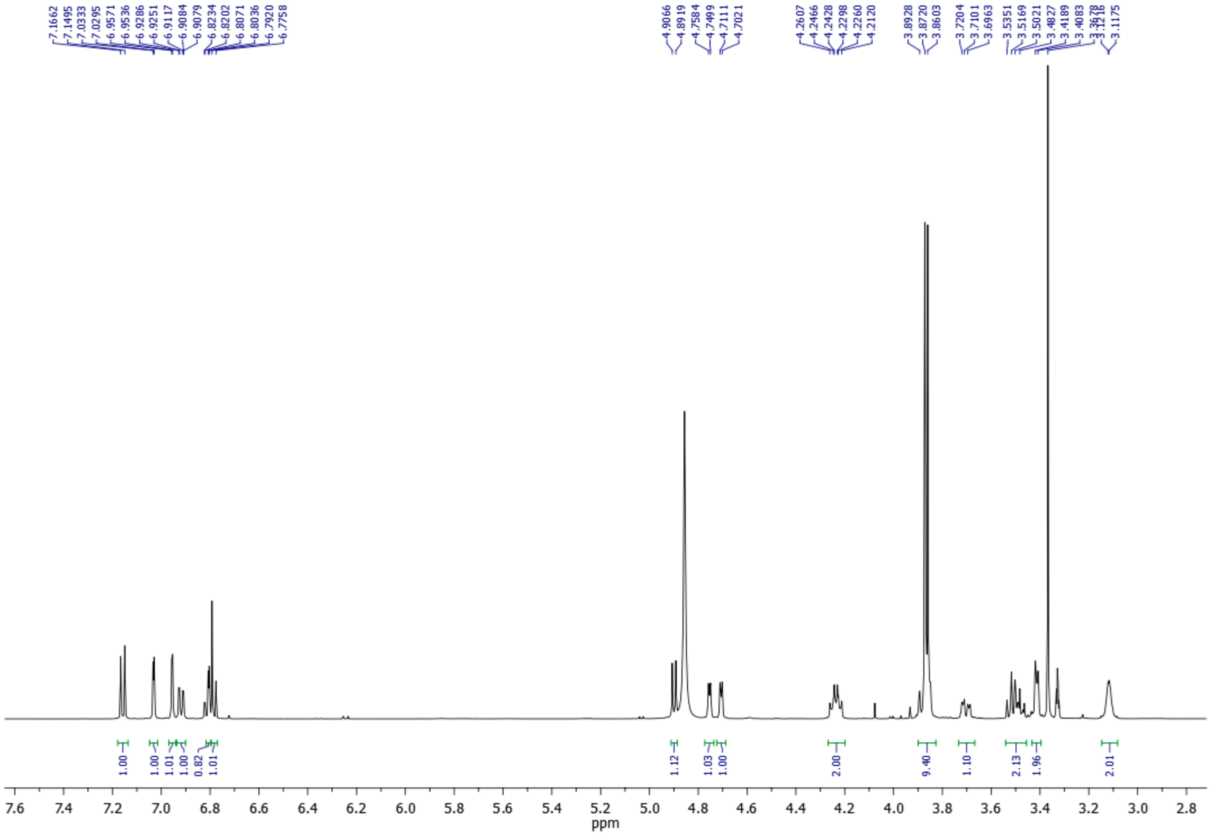

Figure S27. ^{1}H-NMR spectrum of compound **9** (500 MHz, CD_3OD).

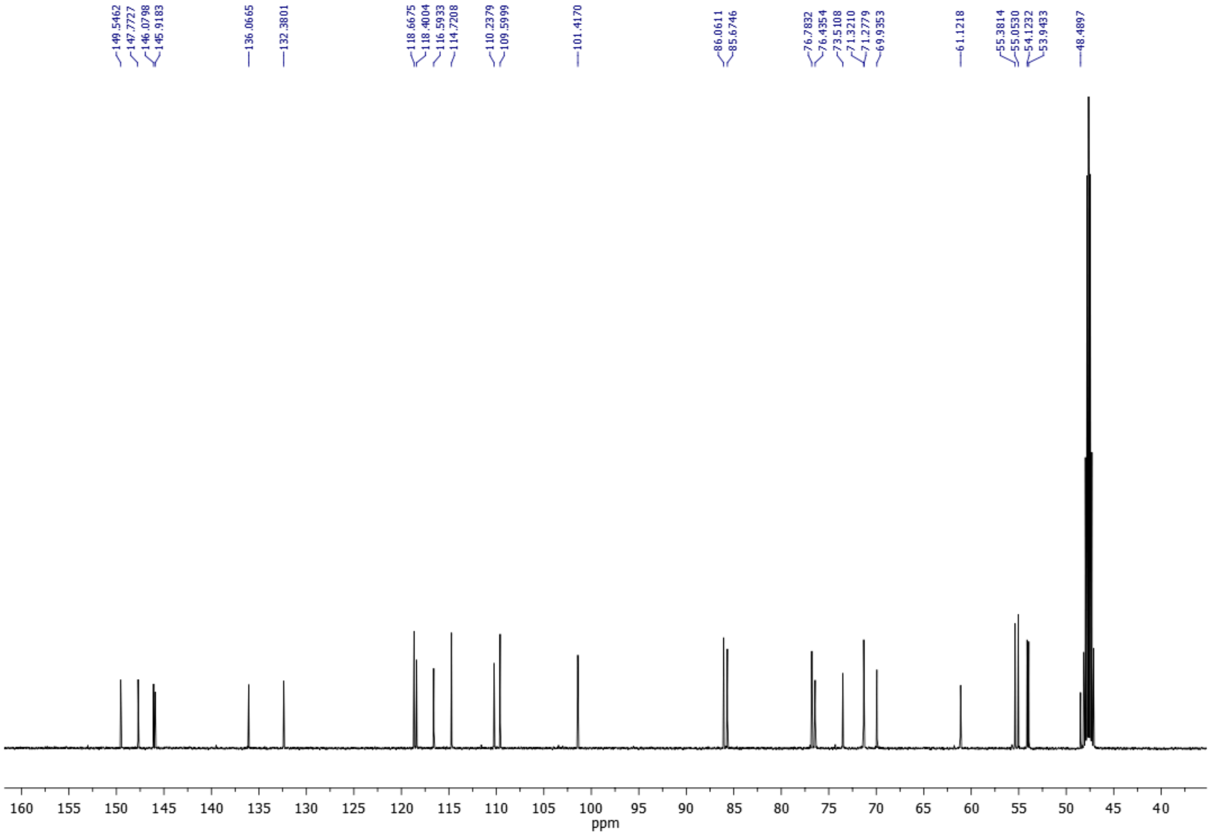

Figure S28. ^{13}C-NMR spectrum of compound **9** (125 MHz, CD_3OD).

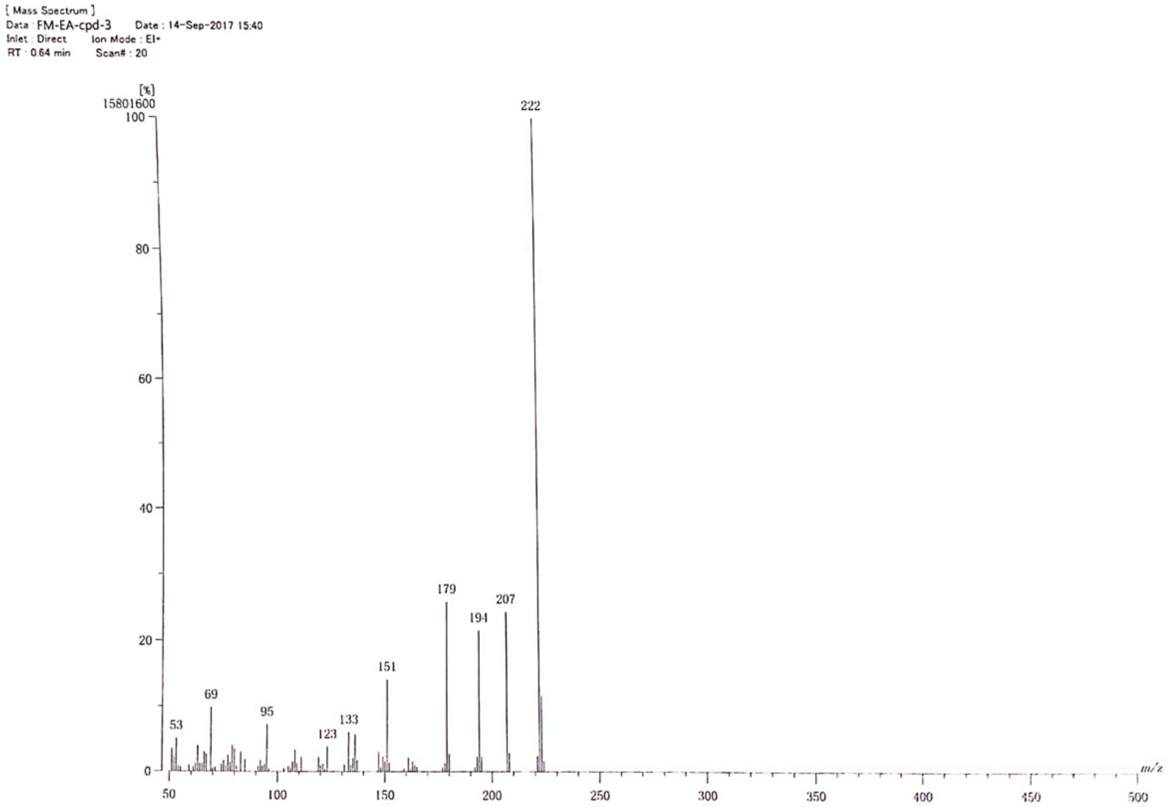

Figure S29. EI-MS of compound **10** (70 eV).

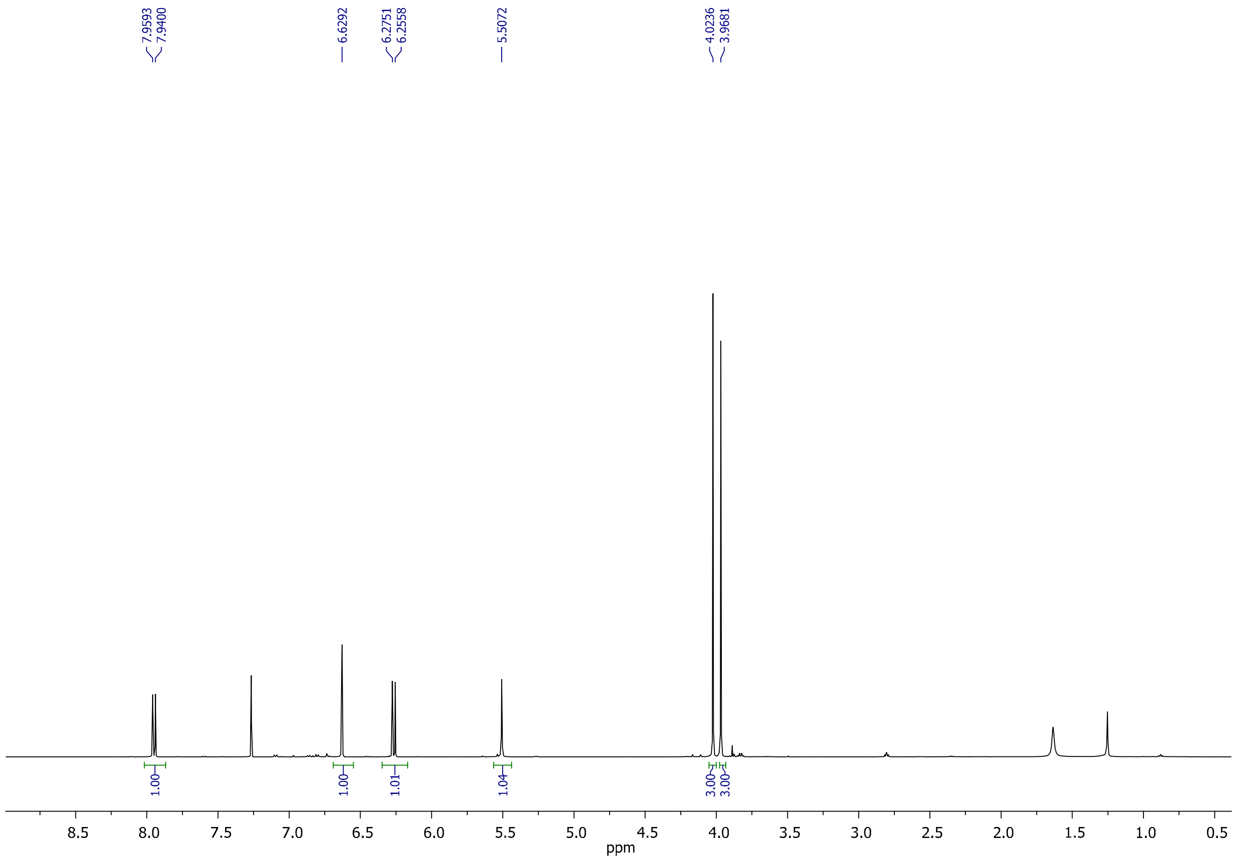

Figure S30. ^{1}H-NMR spectrum of compound **10** (500 MHz, $CDCl_3$).

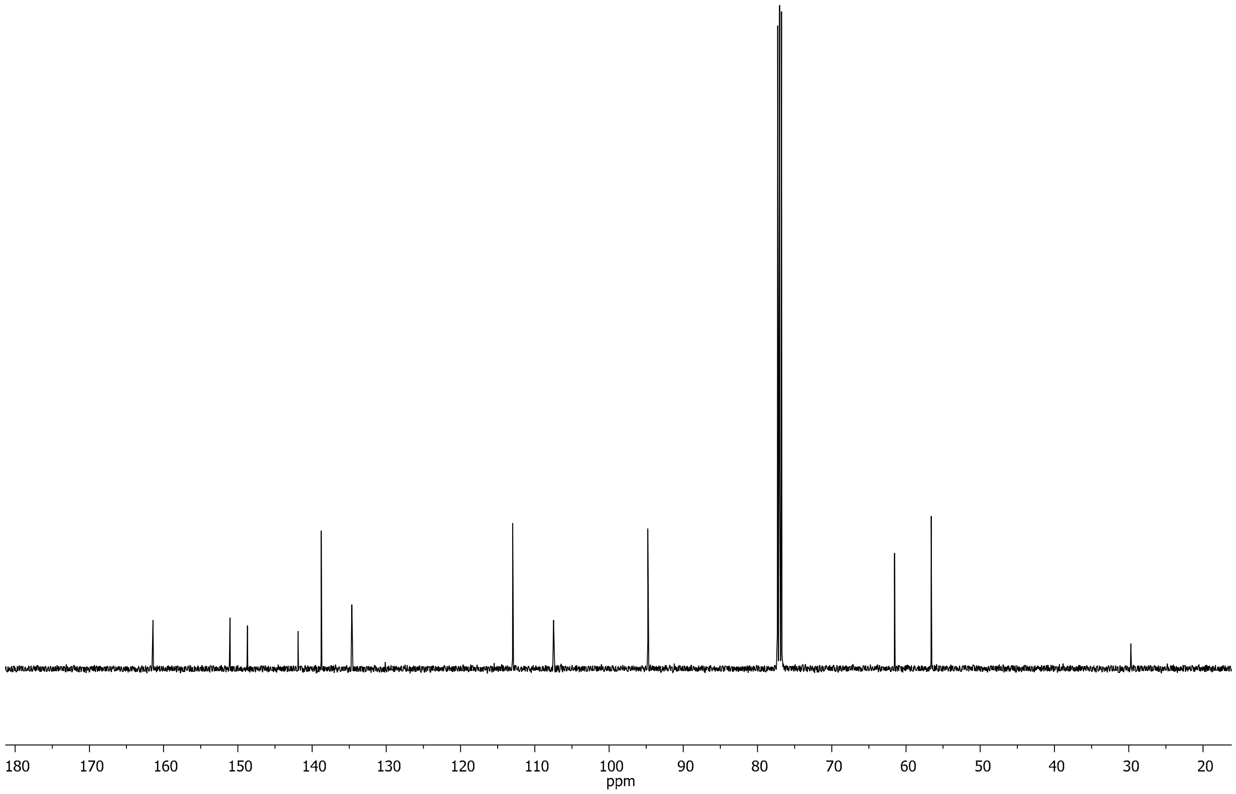

Figure S31. ^{13}C-NMR spectrum of compound **10** (125 MHz, $CDCl_3$).

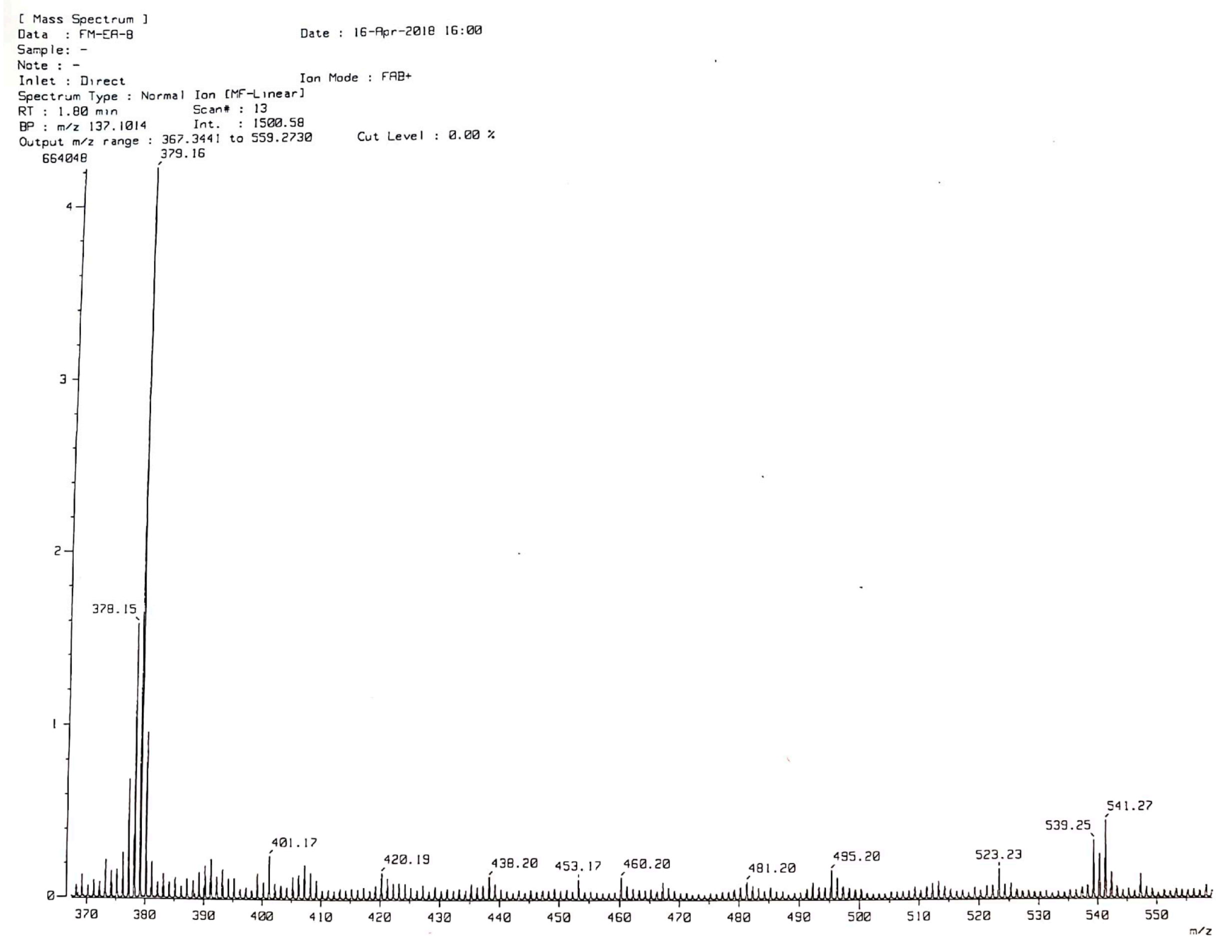

Figure S32. FAB-MS of compound **11** (*m*-NBA).

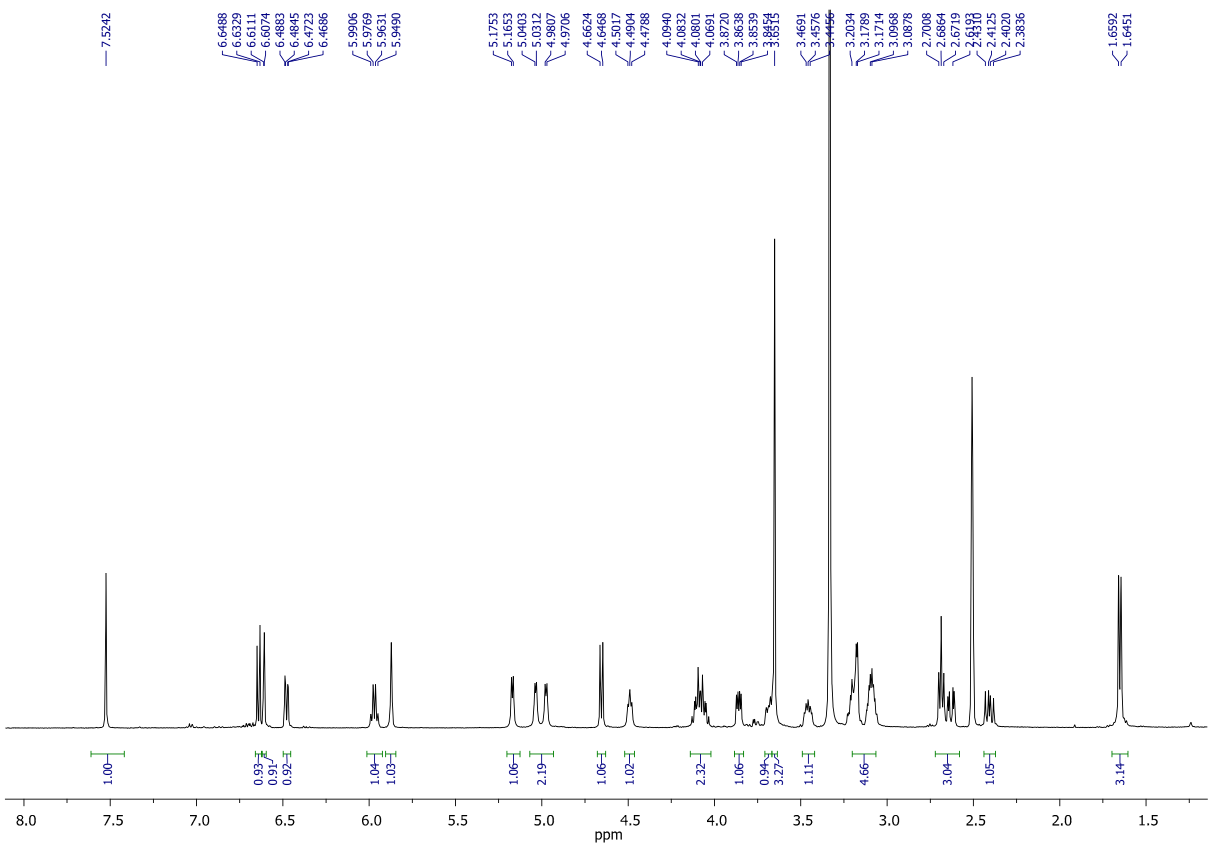

Figure S33. ^{1}H-NMR spectrum of compound **11** (500 MHz, DMSO-d_6).

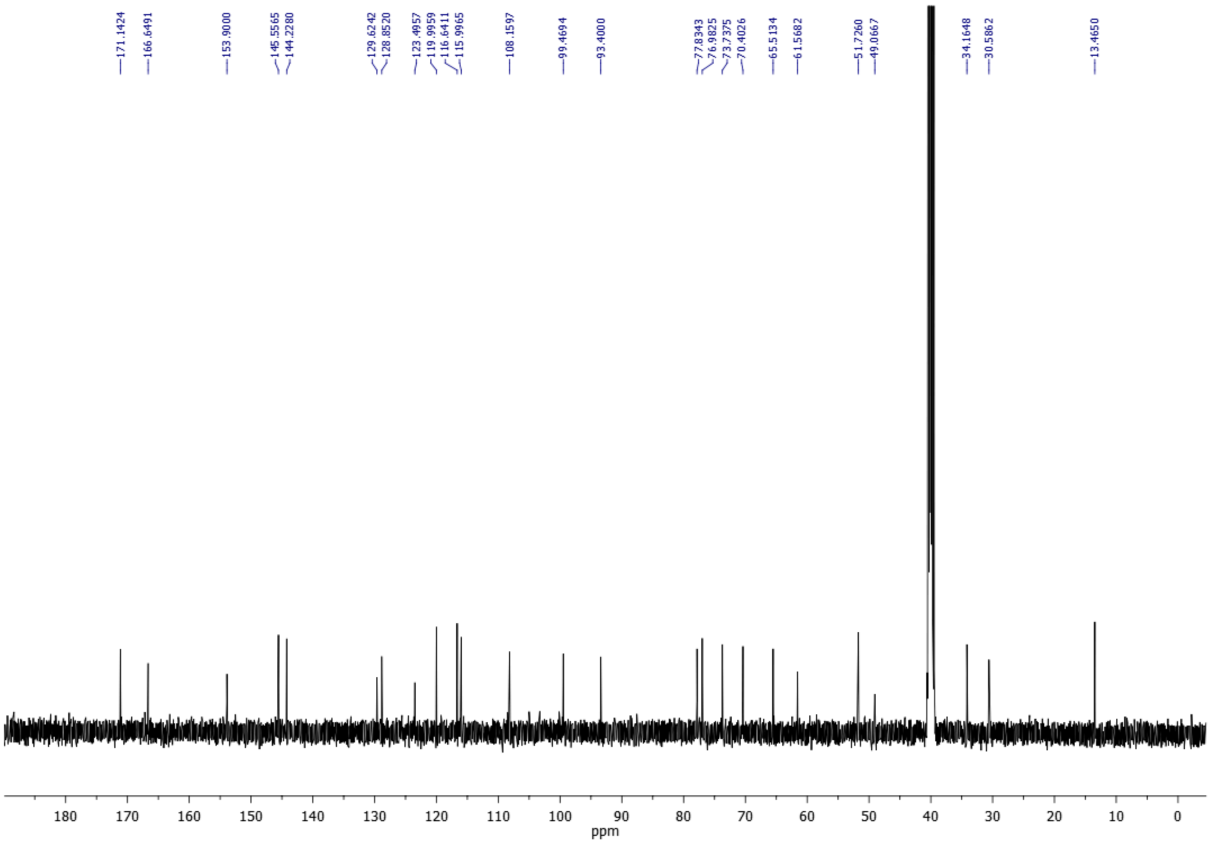

Figure S34. ^{13}C-NMR spectrum of compound **11** (125 MHz, DMSO-d_6).

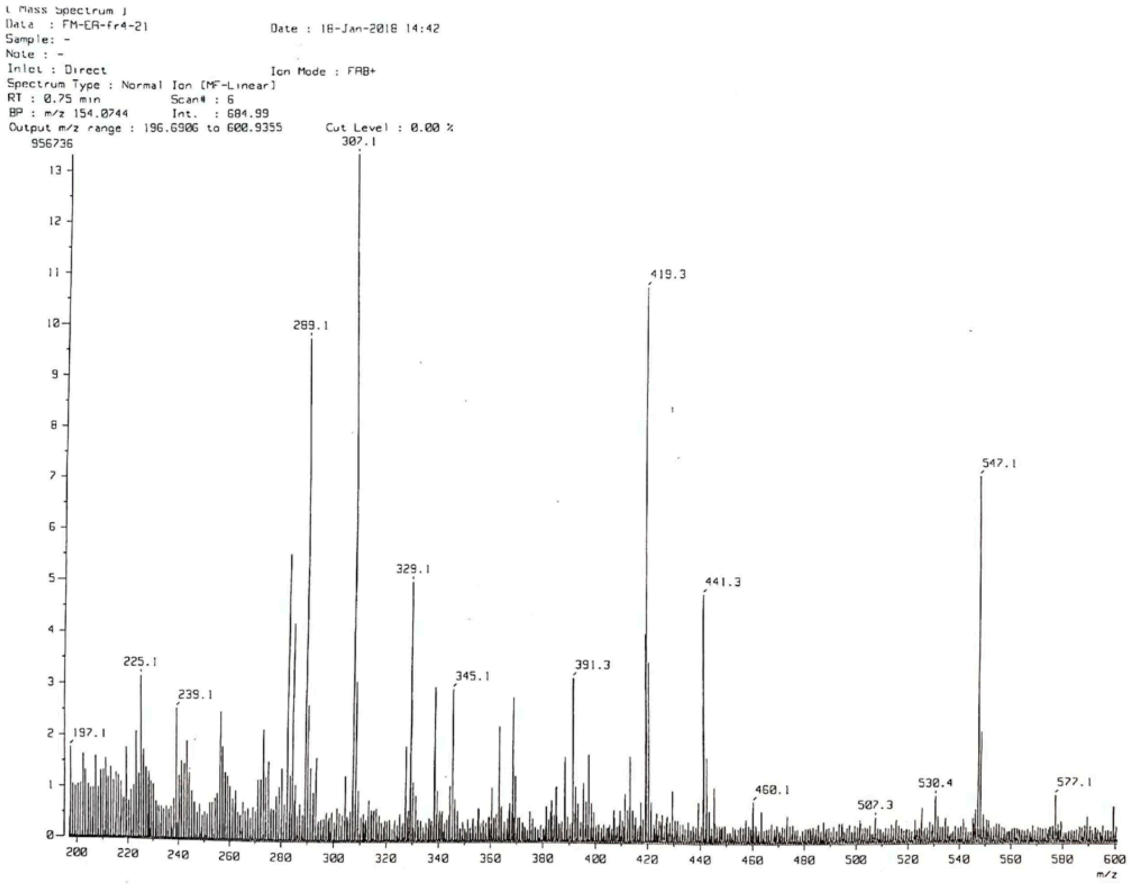

Figure S35. FAB-MS of compound **12** (*m*-NBA).

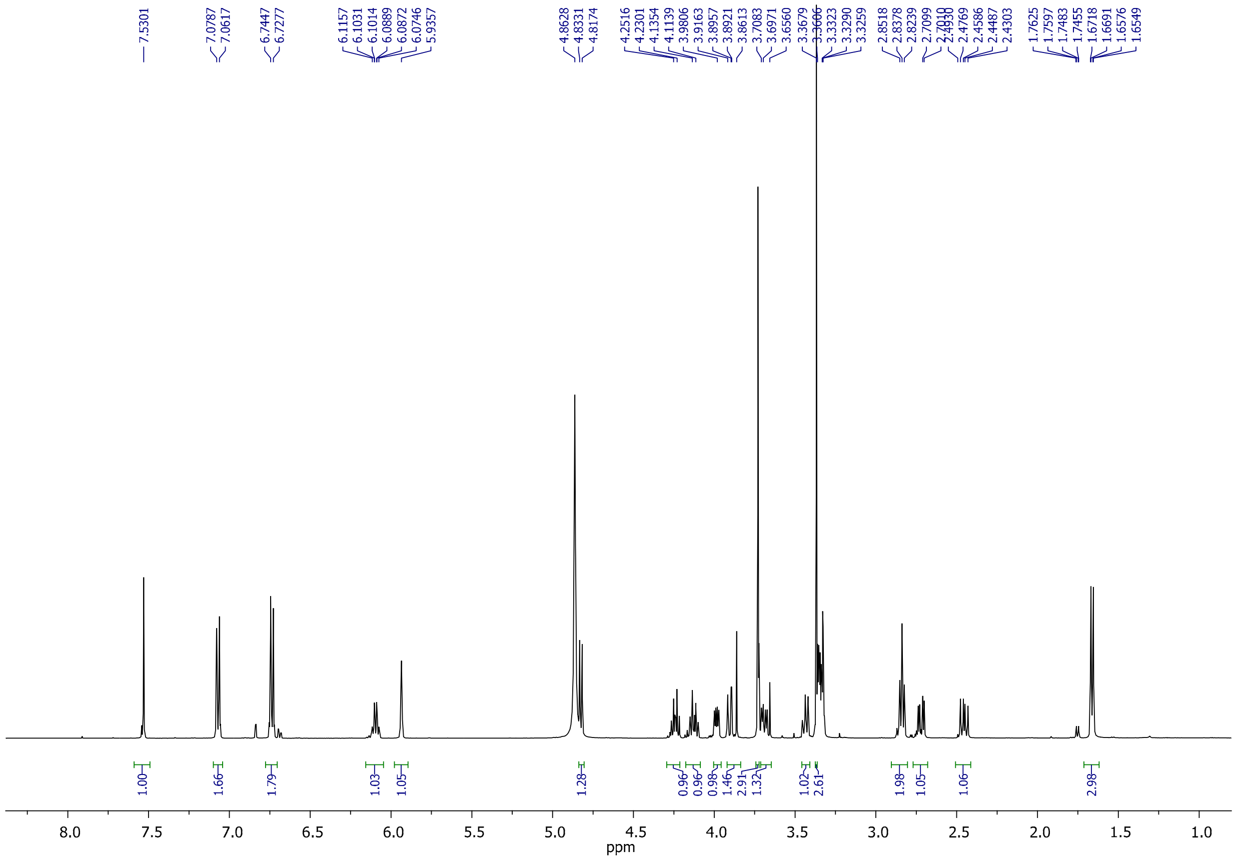

Figure S36. ^{1}H-NMR spectrum of compound **12** (500 MHz, CD_3OD).

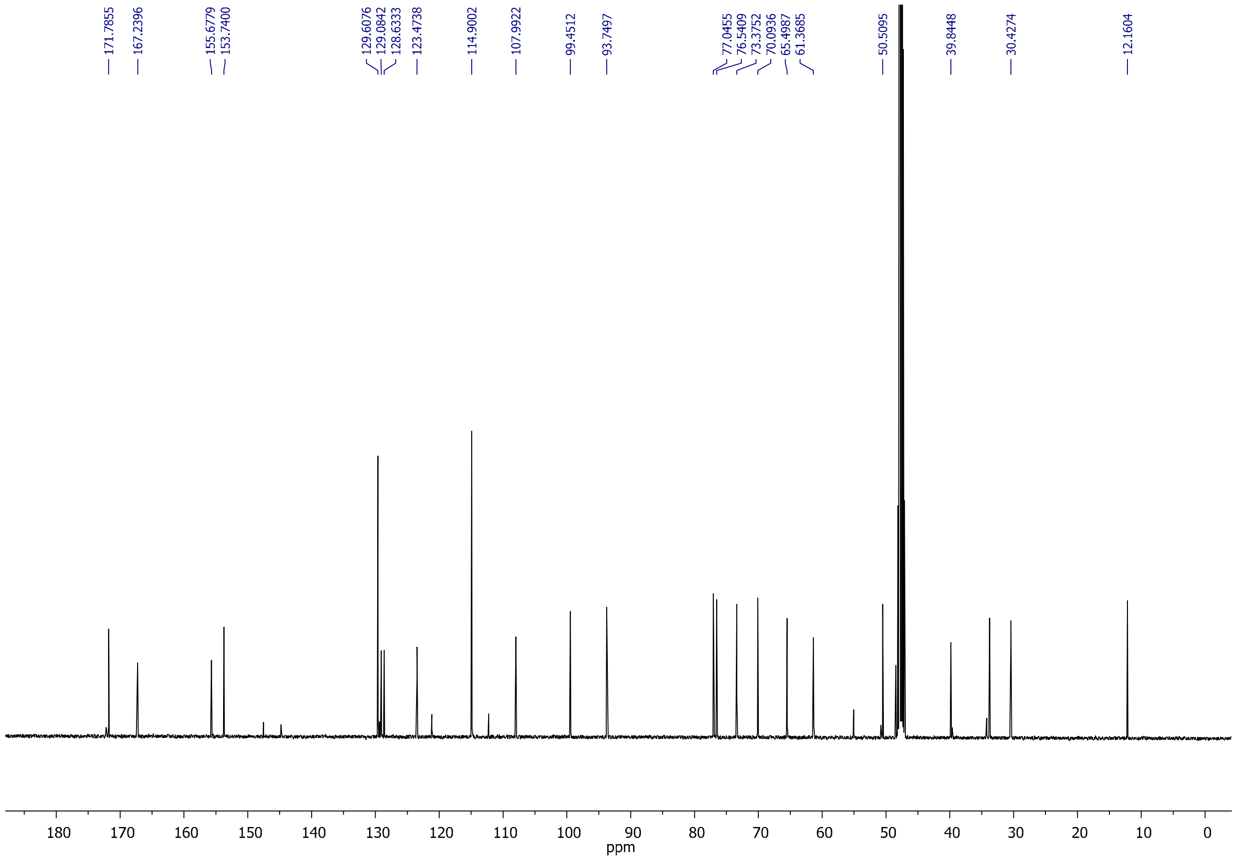

Figure S37. ^{13}C-NMR spectrum of compound **12** (125 MHz, CD_3OD).
